# Electron Affinities from Equation-of-Motion Frozen Pair-Type Coupled Cluster Methods and Their Dependence on Single Excitations, Molecular Orbitals, and Basis Set Sizes

**DOI:** 10.1021/acs.jctc.5c01258

**Published:** 2025-10-06

**Authors:** Saman Behjou, Paweł Tecmer, Katharina Boguslawski

**Affiliations:** Institute of Physics, Faculty of Physics, Astronomy, and Informatics, 317747 Nicolaus Copernicus University in Torun, Grudziądzka 5, Toruń 87-100, Poland

## Abstract

We introduce a series of alternative electron affinity equation-of-motion frozen-pair coupled cluster (EA-EOM-fpCC) methods for computing electron affinities and open-shell electronic structures. These methods are systematically benchmarked against the reference Δ-CCSD­(T) approach and experimental data for a representative molecular data set using natural pair coupled cluster doubles (pCCD) orbitals and various basis set sizes. A comparison to canonical CC methods is also discussed. Additionally, EA-EOM-fpCC results are compared with those derived from the difference between double and single ionization potentials (DIP-EOM-CC and IP-EOM-CC) of dicationic species within the same ground-state fpCC reference framework. Our results demonstrate that frozen-pair approaches significantly reduce computational costs while maintaining high accuracy, offering an efficient strategy for studying electron affinities and open-shell systems in large molecules. The IP/DIP-EOM-fp­(L)­CCSD model stood out as the best post-pCCD flavor to predict EAs, achieving a mean error of 0.09 eV compared to experimental results, while EA-EOM-fpCCD is closest to Δ-CCSD­(T) reference data. Finally, diffuse functions are not recommended for EA calculations using the IP/DIP-EOM-fpCC recipe and are not required for the EA-EOM-fpCC variants if sufficiently large basis sets are employed.

## Graphical abstract

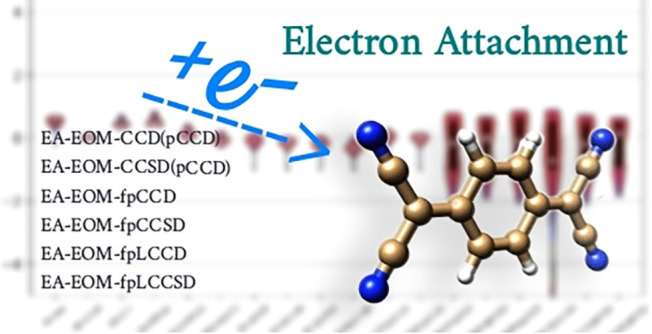

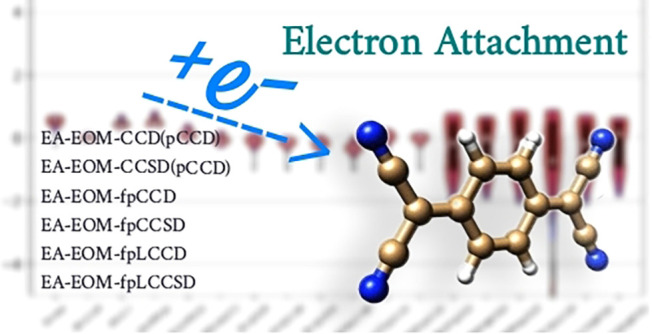

## 1 Introduction

The field of organic electronics^1,2^ is experiencing remarkable growth, driven by the ability to exploit the distinctive properties of organic molecules in devices such as flexible screens, solar panels, sensors, and bioelectronic systems.^3,4^ These organic compounds offer several notable benefits: they can be manufactured inexpensively, exhibit mechanical flexibility, and possess customizable optoelectronic characteristics. The charge transfer process is fundamental to the operation of these devices, which dictates how energy and electrons move between molecular fragments.^5^ As global electricity demand rises, substantial research has focused on creating innovative technologies based on environmentally friendly organic solar cells.^3^ To support such advancements, robust computational methodologies are essential for analyzing the electronic structures and molecular properties of individual components used in these systems.^6^ Significant in this context are the energy levels of the donor’s highest occupied molecular orbital (HOMO) and the acceptor’s lowest unoccupied molecular orbital (LUMO), including the offsets between them.^5,7,8^ These parameters are directly related to the ionization potential and electron affinity of the system.^9–15^

Electronic structure methods such as Hartree–Fock theory and Density Functional Approximations (DFAs)^16–18^ provide a computationally efficient framework to estimate electronic properties for large organic molecules.^6,15,19,20^ However, their accuracy and predictive power is not always reliable. For example, DFAs often struggle to capture strong (nondynamic/static) electron correlation effects, and their overall accuracy strongly depends on the chosen exchange-correlation functional.^14,18,21–23^ Moreover, DFAs face challenges modeling charge-transfer excitations and diffuse anionic states^20,24–26^ which impacts accurate computations of frontier orbital energies, electron affinities, and redox behavior—key factors governing charge-transfer and exciton dynamics in organic electronic devices.^1,3,4,7,25,27–30^ This limitation is especially pronounced for extended π-conjugated systems with small HOMO–LUMO gaps, common components of organic photovoltaic (OPV) materials.^1,13,31^ Despite these shortcomings, quantum chemistry continues to play a vital role in advancing the understanding and prediction of fundamental electronic properties within organic materials.^6,19–21,32–34^

To meet this need, reliable and efficient computational methods are essential for accurately modeling the electronic structures and properties of the fundamental building blocks of OPV materials. Wave function-based approaches have increasingly attracted attention as more robust alternatives, with coupled cluster (CC) theory^35–39^ emerging as particularly promising. Conventional CC methods, such as coupled cluster singles and doubles (CCSD) and CCSD with perturbative triples (CCSD­(T))^40^ can accurately model large molecular systems dominated by dynamic electron correlation effects. O the other hand, simplified CC methods, such as the pair coupled-cluster doubles (pCCD) model^41–46^ offer a cost-effective approach for reliably modeling electronic structures of quasi-degenerate, strongly correlated systems.^47–52^ Combined with an efficient orbital optimization protocol^42–46^ pCCD becomes size consistent. With an addition of suitable dynamic energy corrections^53–57^ on top of the pCCD reference and GPU acceleration,^58^ pCCD-based approaches provide a powerful computational platform for large-scale modeling of electronic structures.^26,42,47,48,51,59–65^

Equation-of-Motion Coupled Cluster (EOM-CC) theory^66,67^ with a frozen pair reference^53^ has been developed to improve the description of open-shell states.^25,68–70^ Despite significant progress on the development and benchmarking of (D)­IP-EOM-pCCD-based methods,^25,52,68,69,71^ their electron-attached analogues remain largely unexplored. Recently, some of us introduced the EA-EOM-pCCD model to target electron-attached states and approximate EAs with a pCCD reference state.^70^ However, for systems with a substantial amount of dynamical correlation, pCCD predicts EAs with a mean error of 1 eV.^70^ To ease the limitations of pCCD, we require robust and efficient recipes to include dynamical correlation, simultaneously allowing the resulting post-pCCD methods to describe electron-attached states. To that end, in this work, we scrutinize the theoretical foundations of various post-pCCD extensions to target electron-attached states and systematically assess their performance for predicting electron affinities in organic molecules.

The structure of this work is as follows: Section 2 offers a concise overview of the investigated theoretical models, while Section 3 details the computational methodology. Numerical results and a statistical analysis are presented in Section 4, with concluding remarks provided in Section 5.

## 2 Theory

### 2.1 Standard Coupled Cluster Models

Canonical coupled cluster methods^35–37^ provide a systematically improvable hierarchy of correlated wave functions through the successive inclusion of higher-order excitation operators,^39,72^ while the exponential ansatz ensures that the CC wave function is size-extensive.^38,73,74^ In this study, we employ both the doubles-only (CCD) and singles-and-doubles (CCSD) variants. The CCD model provides a correlated wave function by accounting for all possible double excitations from a single-reference determinant. It is one of the simplest yet nontrivial approximations in the coupled cluster hierarchy. The CCD wave function is written as

1 $$\left|\Psi_{\mathrm{CCD}}\right\rangle =e^{T_{2}}\left|\Phi_{0}\right\rangle$$

where |Φ_0_⟩ denotes the Hartree–Fock reference determinant, and *T*_2_ is the cluster operator that contains all double excitations. In this work, we employ the spin-free or spin-summed CC framework, where the CCD cluster operator, expressed in terms of spatial orbitals and singlet excitation operators, is given by,^37,72,75^

2 $$T_{2}=\frac{1}{2}\underset{ijab}{\sum }t_{ij}^{ab}E_{a}^{i}E_{b}^{j}$$

with the singlet excitation operator $E_{a}^{i}$ defined as

3 $$E_{a}^{i}=a_{a↑}^{†}a_{i↑}+a_{a↓}^{†}a_{i↓}$$

Here, *i*, *j* index occupied orbitals and *a*, *b* index virtual orbitals. The operator $a_{p}^{†}$ (*a*_*q*_) creates (annihilates) an electron with α (↑) or β (↓) spin in orbital *p* (*q*), the amplitudes $t_{ij}^{ab}$ are obtained by solving a set of nonlinear equations arising from the projection of the similarity-transformed Schrödinger equation onto the space of doubly excited configurations.

By incorporating both single and double excitations, CCSD offers a favorable balance between accuracy and computational cost, enabling accurate treatment of dynamical correlation in a wide variety of systems, including closed-shell molecules, radicals, and weakly correlated species.^39^ The CCSD wave function is defined as

4 $$\left|\Psi_{\mathrm{CCSD}}\right\rangle =e^{T_{1}+T_{2}}\left|\Phi_{0}\right\rangle$$

where the cluster operators *T*_1_ and *T*_2_ generate all possible single and double excitations, respectively. The single excitation operator *T*_1_ takes the form

5 $$T_{1}=\underset{ia}{\sum }t_{i}^{a}E_{a}^{i}$$

where $t_{i}^{a}$ are the corresponding single excitation amplitudes. Restricting the CC ansatz to single and double excitations improves accuracy for a wide range of properties, particularly when describing response functions, excited states, and open-shell systems.^39,72,75–77^ However, standard CCSD breaks down for systems with strong electron correlation.^78,79^

### 2.2 Noncanonical Coupled Cluster Models: pCCD and Post-pCCD

Recently, alternative single-reference CC models have been introduced to capture some degree of strong (static) correlation without resorting to a multireference description. These approaches aim to reduce the complexity of the CC ansatz while retaining essential correlation effects.^80^ One notable example is the pair Coupled Cluster Doubles (pCCD) method,^41–43,50^ which provides a simplified yet effective framework for describing strongly correlated electron pairs.^47,49^ Other examples that can be rewritten using a single-reference CC ansatz are the Antisymmetrized Product of Strongly orthogonal Geminals^81,82^ the Generalized Valence Bond (GVB)^83,84^ ansatz, and their orbital optimized variants.^50,85^ The pCCD method represents a simplified form of the traditional CCD approach, where the *T*_2_ operator of the conventional CCD wave function ansatz of eq 1 is replaced by the electron-pair cluster operator, defined as

6 $$T_{p}=\underset{ia}{\sum }t_{i\mathrm{i̅}}^{a\mathrm{a̅}}a_{a}^{†}a_{\mathrm{a̅}}^{†}a_{\mathrm{i̅}}a_{i}$$

The *T*_p_ operator promotes electron pairs from occupied to virtual orbitals. The coefficients $t_{i\mathrm{i̅}}^{a\mathrm{a̅}}$ correspond to the pCCD cluster amplitudes, where indices *i*, *a* label α/↑ spin degrees of freedom, while *i̅*, *a̅* encode the β/↓ spin block. The reference wave function |Φ_0_⟩ is an independent-particle state. A typical starting point is the Hartree–Fock (HF) determinant. However, the reference state is optimized during an orbital-optimized pCCD calculation,^42–46^ which recovers size consistency. By focusing solely on pair excitations, pCCD efficiently captures the essential static correlation effects with reduced computational effort.^49^ On the other hand, it neglects dynamic electron correlation effects arising from broken-pair (seniority nonzero) excitations. To remedy this limitation, various coupled cluster corrections have been developed on top of the pCCD wave function to recover the missing dynamic electron correlation.^53,54,57^

In particular, the *frozen-pair* coupled cluster (fpCC) approach employs a factorized exponential ansatz,^57,86–95^ where the pCCD wave function |Ψ_pCCD_⟩ is taken as a fixed reference and an additional cluster operator accounts for all nonpair excitations. The fpCCD wave function is thus defined as

7 $$\left|\Psi_{\mathrm{fpCC}}\right\rangle =e^{T_{2}^{\prime }}\left|\Psi_{\mathrm{pCCD}}\right\rangle =e^{T_{2}^{\prime }}e^{T_{p}}\left|\Phi_{0}\right\rangle ,\;\mathrm{with}\;T_{2}^{\prime }=T_{2}-T_{p}$$

where $T_{2}^{\prime }$ is the broken-pair doubles operator containing all seniority nonzero double excitations that are *absent* from the pCCD operator. Furthermore, the amplitudes in *T*_p_ are typically *frozen* at values obtained from the pCCD calculation, and only the broken-pair amplitudes in $T_{2}^{\prime }$ are optimized to recover dynamic electron correlation missing in pCCD.^53,57^ Thus, fpCC theory can be understood as a variant of tailored CC theory,^57,86–95^ where the multireference nature of quantum states is approximated by a pCCD wave function. The frozen-pair CCSD (fpCCSD) ansatz also includes single excitations in the broken-pair cluster operator,

8 $$\left|\Psi_{\mathrm{fpCCSD}}\right\rangle =e^{T_{1}+T_{2}^{\prime }}\left|\Psi_{\mathrm{pCCD}}\right\rangle$$

Alternatively, the fpCC ansatz has also been simplified by truncating the Baker–Campbell–Hausdorff (BCH) expansion and neglecting all nonlinear terms in the broken-pair amplitudes.^54^ This yields a linearized version of the pCCD-tailored theory known as frozen-pair linearized coupled cluster (fpLCC) or pCCD-LCC. These linearized models provide efficient, low-scaling post-pCCD corrections that capture the leading-order dynamic electron correlation effects.^62,65,96^ We should note that, strictly speaking, fpLCC does not fall into the category of tailored coupled cluster methods, yet it offers a computationally efficient and systematically improvable approach to incorporate dynamical correlation on the pCCD reference.^56,57^ Specifically, the fpLCCD wave function is approximated as

9 $$\left|\Psi_{\mathrm{fpLCCD}}\right\rangle \approx \left(1+T_{2}^{\prime }\right)\left|\Psi_{\mathrm{pCCD}}\right\rangle$$

Thus, although the cluster equations are linear with respect to the nonzero seniority amplitudes $T_{2}^{\prime }$ , the coupling between pair and nonpair excitations is fully included (as well as disconnected *T*_p_ terms). Similarly, the fpLCCSD wave function can be approximated as

10 $$\left|\Psi_{\mathrm{fpLCCSD}}\right\rangle \approx \left(1+T_{1}+T_{2}^{\prime }\right)\left|\Psi_{\mathrm{pCCD}}\right\rangle$$

This method introduces both single excitations and nonpair double excitations on top of the pCCD reference. The inclusion of *T*_1_ accounts for orbital relaxation, while $T_{2}^{\prime }$ captures the dominant part of dynamic electron correlation. Both fpCC methods and their linearized variants have been employed to study molecules and their properties in their electronic ground states, including organic compounds and complexes containing transition metals, lanthanides, and actinides.^26,48,51,54,57,59–65,97^

### 2.3 Equation-of-Motion Coupled Cluster Theory

Equation-of-Motion (EOM-)­CC theory^66^ is a versatile extension of ground-state CC theory that enables the calculation of electronically excited, ionized, electron-attached, and spin-flip states based on a single-reference CC wave function.^67,75,77,98–102^ In the EOM framework, the target state |Ψ_*k*_⟩ is generated by applying a linear excitation operator *R*_*k*_ to the correlated CC ground-state wave function,

11 $$\left|\Psi_{k}\right\rangle =R_{k}e^{T}\left|\Phi_{0}\right\rangle$$

The excitation energies ω_*k*_ are the eigenvalues of the non-Hermitian similarity-transformed Hamiltonian *H̅*

12 $$\mathrm{H̅}R_{k}\left|\Phi_{0}\right\rangle =\omega_{k}R_{k}\left|\Phi_{0}\right\rangle ,,\mathrm{H̅}=e^{-T}He^{T}$$

where ω_*k*_ = *E*_*k*_ – *E*_0_ are the excitation, ionization, or attachment energies relative to the ground-state energy *E*_0_. By changing the ansatz of *R*_*k*_, EOM-CC can target states with different particle numbers or spin multiplicities. Furthermore, EOM-CC methods preserve size-intensivity (for excitation energies), and their accuracy can be systematically improved by including higher-rank excitations in both *T* and *R*_*k*_.^103–107^

In this work, we derive, implement, and investigate different EOM formalisms to describe electron-attached states. Specifically, these include the Electron-Affinity (EA) variant^98,101^ of EOM and electron affinities approximated by the energetic difference between ionized and doubly ionized states described by the Ionization Potential (IP) and Double Ionization Potential (DIP) flavors of EOM-CC theory.^98–100^ Furthermore, when pursuing EAs calculations within the EA-EOM-CC framework, two principal choices for the excitation operator ansatz can be considered: a spin-integrated or a spin-free (spin-summed) formulation.^25,69,108,109^ In the spin-integrated approach, the explicit spin-dependence of both the excitation operator *R* and the corresponding projection manifold is retained, enabling the selective targeting of states with specific spin projections *S*_*z*_. This approach provides flexible access to a broad manifold of spin states (e.g., doublet, quartet, sextet, etc.) depending on the choice of *R* and reference. In particular, high-spin EOM-CC variants—such as those employing |*S*_*z*_|>1/2—allow significant simplifications of the working equations and efficient computations of high-spin states.^108–110^ Alternatively, in the spin-free version, a spin summation is performed to remove explicit spin-dependence, so that only “spin-free doublet” states are directly targeted in the EOM procedure.^109^

### 2.4 EA-EOM-fp­(L)­CC Formalism and Diagrammatic Structure

In this work, we implemented and benchmarked the *S*_*z*_ = −1/2 variant of EA-EOM-CC theory for various post-pCCD reference states. Specifically, the electron attachment operator $R_{k}^{\mathrm{EA}}$ is restricted to the *S*_*z*_ = −1/2 states^109^ and at most two particle and one hole operators,

13 $$R_{S_{z}=-1/2}^{\mathrm{EA}}=\underset{a}{\sum }r^{a}a_{a}^{†}+\frac{1}{2}\underset{abj}{\sum }r_{j}^{ab}a_{a}^{†}a_{b}^{†}a_{j}+\underset{a\mathrm{b̅}\mathrm{j̅}}{\sum }r_{\mathrm{j̅}}^{a\mathrm{b̅}}a_{a}^{†}a_{\mathrm{b̅}}^{†}a_{\mathrm{j̅}}$$

In this case, the configurational space used in the diagonalization of eq 12 is spanned by determinants of the form |Φ^*a*^⟩, $\left|\Phi_{j}^{ab}\right\rangle$ , $\left|\Phi_{\mathrm{j̅}}^{a\mathrm{b̅}}\right\rangle$ , corresponding to one particle and two-particle-one-hole excitations, respectively.^70^ The working equations in the diagrammatic representation of the EA-EOM-fp­(L)­CCSD equations are shown in Figure 1, while the algebraic equations are summarized in the Supporting Information. The EA-EOM-fp­(L)­CCSD equations are structurally similar to those in the conventional EA-EOM-CCSD framework. Note, however, that the EA-EOM-fpLCC­(S)­D equations omit selected disconnected *T*_1_ terms. Specifically, all effective Hamiltonian diagrams (a1) and (b2) to (b5) omit all disconnected *T*_1_ terms involving nonpair excitations, that is, *T*_1_ or (broken-pair) $T_{2}^{\prime }$ . Moreover, diagram (b1) lacks the disconnected $T_{1}^{3}$ (*T*_1_ *T*_1_ *T*_1_) contribution and instead contains a simplified *T*_1_ *T*_p_ component, which arises due to the truncation of the BCH-expansion in the LCC treatment of nonpair excitations only (see also eq 10, which includes *T*_1_ *T*_p_ terms). The corresponding modifications of the (b1) terms are shown in Figure 2 and present the surviving disconnected diagrammatic contributions of the EA-EOM-fpLCCSD formalism. Note that the *T*_2_ vertex represents the *T*_p_ excitation operator. The labels β, γ, and δ denote the spin degree of freedom of the corresponding *T* vertex and the spin block of the corresponding *R* operator. Typically, β refers to a beta-spin orbital, while γ and δ represent general spin orbitals. Since the spin degrees of freedom of *T*_p_ are defined by the corresponding open lines (*T*_p_ contains a mixed-spin component or α - β block), only one term survives for the $R_{j}^{ab}$ part, while all four diagrams contribute to the $R_{\mathrm{j̅}}^{a\mathrm{b̅}}$ equations.

**Figure 1:**
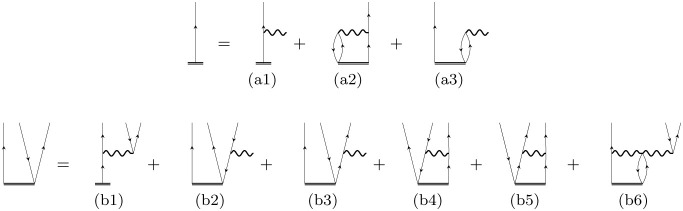
Diagrammatic representation of the EA-EOM-fp­(L)­CCSD equations (antisymmetrized formalism). The top line corresponds to the first set of diagrams (a1–a3), and the bottom line corresponds to the second set (b1–b6). See the Supporting Information for corresponding algebraic expressions.

**Figure 2:**
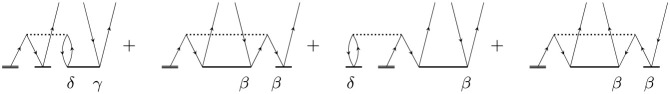
Diagrammatic representation of the surviving disconnected terms in (b1) shown in Figure 1 of the EA-EOM-fpLCCSD model (Goldstone formalism) involving contractions between the single excitation operator *T*_1_ and the electron-pair excitation operator *T*_p_. For $R_{j}^{ab}$ , only the first diagram contributes, where γ = α and δ = β. For the $R_{\mathrm{j̅}}^{a\mathrm{b̅}}$ part, all diagrams enter with γ = β and δ = α (first diagram) and δ = α, β (third diagram). Note that all unlabeled lines correspond to α spin. See the Supporting Information for corresponding algebraic expressions.

### 2.5 EAs from IP/DIP-EOM-CC Methods

An alternative approach to describing EAs relies on approximating them via ionization processes using the IP and DIP variants of EOM-CC methods. These IP-EOM and DIP-EOM approaches provide a powerful framework to access electron-attached states indirectly and have been successfully applied in recent works.^52,70^ Having access to the ionization energies *E*_IP_ and double-ionization energies *E*_DIP_, the electron affinity can be estimated as

14 $$E_{\mathrm{EA}}=E_{\mathrm{DIP}}-E_{\mathrm{IP}}$$

To evaluate this expression, both the single and double ionization energies must be computed from the same (possibly negatively charged) reference state. Specifically, we start from an (*N* + 2)-electron reference and employ DIP-EOM and IP-EOM to determine the energies of the *N*- and (*N* + 1)-electron states, respectively. The energy of the (*N* + 1)-electron system is obtained by applying the IP-EOM ansatz to the (*N* + 2)-electron reference state, while the DIP-EOM operator generates the *N*-electron states from the same starting point. This allows EAs to be estimated as the energy gap between these two ionized states. Furthermore, we will restrict the *R* operator of the IP and DIP variant to contain at most two hole and one particle (2h1p) and three hole and one particle (3h1p) terms, respectively. The EAs are then determined from

15 $$E^{\mathrm{EA}}=E_{0}^{\mathrm{DIP}}\left[3h,1p\right]-E_{0}^{\mathrm{IP}}\left[2h,1p\right]$$

where $E_{0}^{\mathrm{IP}}\left[2h,1p\right]$ and $E_{0}^{\mathrm{DIP}}\left[3h,1p\right]$ denote the (doublet or singlet) ground-state (0-th root) energies obtained from the IP-EOM-CC and DIP-EOM-CC formalisms involving at most two-hole-one-particle and three-hole-one-particle excitations, respectively.

Similar to the EA-EOM-fp­(L)­CC formalism described above, we target *S*_*z*_ = −1/2 (doublet and quartet) states in IP-EOM-CC calculations and *S*_*z*_ = 0 (singlet, triplet, and quintet) states in DIP-EOM-CC ones. The corresponding *R* operators take on the form

16 $$R_{S_{z}=-1/2}^{\mathrm{IP}}=\underset{i}{\sum }r_{i}a_{i}+\frac{1}{2}\underset{ija}{\sum }r_{ij}^{a}a_{a}^{†}a_{j}a_{i}+\underset{i\mathrm{j̅}\mathrm{a̅}}{\sum }r_{i\mathrm{j̅}}^{\mathrm{a̅}}a_{\mathrm{a̅}}^{†}a_{\mathrm{j̅}}a_{i}$$

and

17 $$R_{S_{z}=0}^{\mathrm{DIP}}=\underset{i\mathrm{j̅}}{\sum }r_{i\mathrm{j̅}}a_{\mathrm{j̅}}a_{i}+\frac{1}{2}\underset{i\mathrm{j̅}ka}{\sum }r_{i\mathrm{j̅}k}^{a}a_{a}^{†}a_{k}a_{\mathrm{j̅}}a_{i}+\frac{1}{2}\underset{i\mathrm{j̅}\mathrm{k̅}\mathrm{a̅}}{\sum }r_{i\mathrm{j̅}\mathrm{k̅}}^{\mathrm{a̅}}a_{\mathrm{a̅}}^{†}a_{\mathrm{k̅}}a_{\mathrm{j̅}}a_{i}$$

where the first term corresponds to the removal of a single (or two) electron(s) from an occupied orbital *i* (and *j*), and the higher-order terms describe shakeup excitations that account for simultaneous electron removal and excitations of other electrons. The corresponding IP- and DIP-EOM-fp­(L)­CC models are scrutinized in refs. 52,69 Finally, some of us have shown that these frozen-pair-type variants allow us to approximate EAs that are in good agreement with experimental data in organic systems featuring some degree of strong correlation.^52^ In this work, we aim to compare the DIP/IP recipe to predict EAs with the EA-EOM-CC flavors and study their dependence on the basis set.

### 2.6 Notes on Scaling

The formal computational scaling of EA-EOM-fpCC-based methods is similar to the conventional EA-EOM-CCD/EA-EOM-CCSD flavors. While the evaluation of the CC ground-state amplitude equations (namely, the vector function) has a computational cost of $O\left(o^{2}v^{4}\right)$ , where *o* is the number of occupied and *v* the number of virtual orbitals, respectively, one matrix-vector multiplication of the effective Hamiltonian with a trial vector of a Davidson iteration step formally scales as $O\left(ov^{4}\right)$ (using suitable intermediates). For the (D)­IP-EOM-CC counterparts, the computational cost of solving the EOM equations reduces to $O\left(o^{3}v^{2}\right)$ (IP) or $O\left(o^{4}v^{2}\right)$ (DIP), respectively. Thus, the computational bottleneck of all EA-EOM-CC and (D)­IP-EOM-CC approaches is given by the ground-state calculation of $O\left(o^{2}v^{4}\right)$ cost, while CCSD­(T) formally scales as $O\left(N^{7}\right)$ (*N* = *o* + *v*).

## 3 Computational Details

All the implementations and quantum chemical calculations are performed in a developer version of the PyBEST software package (v2.0.0-dev0).^111,112^ Multiple excitation flavors based on fpCCD and fpCCSD and their linearized variants (fpLCCD and fpLCCSD) are investigated, resulting in the EA-EOM-fpCCD, EA-EOM-fpCCSD, EA-EOM-fpLCCD, and EA-EOM-fpLCCSD methods. A detailed description of each EA-EOM-fpCC model is provided in Subsection [Sec sec2.4]. Additionally, the EAs are also calculated as energy differences between the doubly ionized (DIP-EOM-fpCC) and singly ionized (IP-EOM-fpCC) energies of doubly charged reference ground states according to eq 15.

In all our calculations, we used the Cholesky decomposition^113^ of the two-electron integrals (threshold of 10^–4^) and a frozen core approximation for correlated calculations as implemented in PyBEST. GPU-acceleration was employed using the CuPy library^114^ as supported in PyBEST.^58^ Alternatively, to further accelerate EOM-CC computations, density-fitting (DF) techniques and their combination with Cholesky decomposition (CD) have been reported previously, including efficient treatments of particle–particle ladder terms.^115,116^

In addition, we verified the effect of the CD threshold on total and relative energies. Specifically, we performed test calculations for two CD thresholds (10^–4^ and 10^–5^), two basis sets (aug-cc-pVDZ and cc-pVTZ), and fpCCSD-based methods (pCCD, fpCCSD, EA-EOM-fpCCSD, DIP/IP-EOM-fpCCSD). In general, relative energies (like EAs or DIP/IPs) are less affected by decreasing the CD threshold. Errors in EAs are typically below 0.001 eV, while the total energies of the fpCCSD reference state change less than 0.001 *E*_*h*_. Thus, the EAs are essentially unaffected by reasonably small CD thresholds if the orbitals are optimized within pCCD. Throughout this work, we chose a CD threshold of 10^–4^ for all basis sets. The corresponding numerical results are summarized in the Supporting Information Table S10.

We employed the variationally optimized pCCD natural orbitals^42^ in combination with the cc-pVDZ, aug-cc-pVDZ, and cc-pVTZ basis sets.^117,118^ All the EOM-fpCC methods used only the pCCD orbitals, while the EOM-CC methods used both canonical HF orbitals and pCCD orbitals, denoted as EA-EOM-CC and EA-EOM-CC­(pCCD), respectively. Based on these calculations, we performed the complete basis set (CBS) limit estimation of total energies using the two-point extrapolation formula^119,120^

18 $$E_{X}=E_{\mathrm{CBS}}+aX^{-3}$$

In the above equation, X is the cardinal number of the atomic basis set (X = 2 for cc-pVDZ, X = 3 for cc-pVTZ), *E*_*X*_ is the corresponding total energy of a specific state, and *a* is some fitted parameter.

### 3.1 The Molecular Data Set

The accuracy of our methods is benchmarked against a set of 24 organic molecules depicted in Figure 3, for which xyz structures are available.^121,122^ The theoretical and experimental reference data for EAs are taken from refs 121,123 All molecular structures shown in Figure 3 were rendered using the Jmol molecular visualization software.^124^

**Figure 3:**
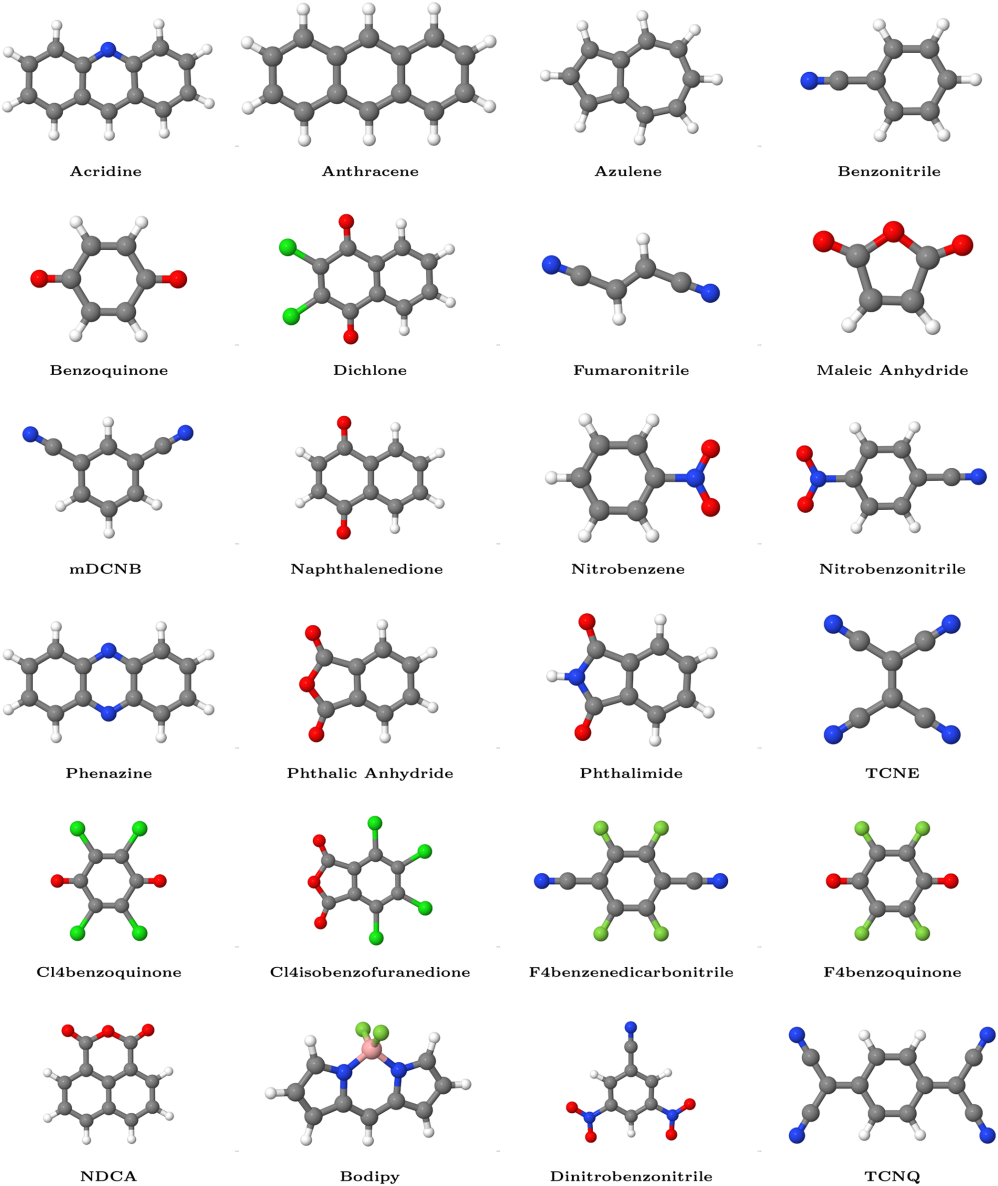
Molecular structures of the 24 acceptor molecules investigated in this work. The molecular coordinates were taken from refs 121,122 and rendered using the Jmol molecular visualization software.^124^ Note that experimental EA values are unavailable for Bodipy and NDCA.

### 3.2 Statistical Analysis

The performance of various methods is assessed using the following statistical metrics: Mean Error (ME), Mean Absolute Error (MAE), Root-Mean-Square Error (RMSE), Mean Percentage Error (MPE), and Standard Deviation (SD) (see SI for more details). All sums run over the molecular test set of *N* = 24 organic acceptor molecules in case of Δ-CCSD­(T) reference data (aug-cc-pVDZ), while only *N* = 22 data points are available for the experimental reference data (see also Supporting Information).

## 4 Results and Discussion

In this section, we present a detailed assessment of EAs computed with various coupled cluster-based methods with a pCCD reference state. Our benchmark set comprises 24 organic acceptor molecules, for which Δ-CCSD­(T)^39,40^ reference values and experimental electron affinities (22 out of 24)^121,123^ are available. Specifically, we scrutinize the quality of EAs predicted by post-pCCD methods exploiting the EA-EOM-CC and DIP/IP-EOM-CC formalisms and their dependence on basis set size, single excitations, and choice of molecular orbitals (localized pCCD orbitals vs delocalized canonical HF ones).

### 4.1 EA-EOM-CC Methods

The upper part of Tables 1 and 2 summarize the statistical errors for EAs computed within the various EA-EOM-fpCC and EA-EOM-CC frameworks and different basis set sizes with respect to the Δ-CCSD­(T) reference (aug-cc-pVDZ) and experiment (three different basis sets and CBS limit). The corresponding EAs are provided in the spreadsheet, while the differences w.r.t. reference data are collected in Tables S1, S3, S5, and S7 of the Supporting Information.

**Table 1:** Statistical Error Metrics (ME, MAE, RMSE, MPE, SD) [eV] for Computed Electron Affinities (EAs) of 24 Organic Acceptor Molecules Using Various Methods and the aug-cc-pVDZ Basis Set, Relative to Δ-CCSD­(T) Reference Values from Refs 121 and 123 ^a^

		Errors w.r.t. Δ-CCSD(T) (*E*_method_ – *E*_ref_)				
Method	Basis Set	ME [eV]	MAE [eV]	RMSE [eV]	MPE [%]	SD [eV]
EA-EOM-fpCCD	aug-cc-pVDZ	–0.11	0.14	0.24	23.8	0.22
EA-EOM-fpCCSD	aug-cc-pVDZ	–0.16	0.16	0.25	26.9	0.20
EA-EOM-fpLCCD	aug-cc-pVDZ	–0.21	0.21	0.29	36.5	0.20
EA-EOM-fpLCCSD	aug-cc-pVDZ	–0.31	0.32	0.37	49.2	0.20
EA-EOM-CCD(pCCD)	aug-cc-pVDZ	0.01	0.13	0.23	18.1	0.24
EA-EOM-CCSD(pCCD)	aug-cc-pVDZ	–0.03	0.09	0.21	13.7	0.22
DIP/IP-EOM-fpCCD	aug-cc-pVDZ	–0.30	0.78	0.90	128.8	0.87
DIP/IP-EOM-fpCCSD	aug-cc-pVDZ	–0.28	0.73	0.85	122.3	0.82
DIP/IP-EOM-fpLCCD	aug-cc-pVDZ	–0.24	0.82	0.92	130.7	0.91
DIP/IP-EOM-fpLCCSD	aug-cc-pVDZ	–0.64	1.16	1.73	147.0	1.65
DIP/IP-EOM-CCD(pCCD)	aug-cc-pVDZ	–0.33	0.77	0.90	128.0	0.85
DIP/IP-EOM-CCSD(pCCD)	aug-cc-pVDZ	–0.27	0.69	0.82	118.1	0.79
EA-EOM-CCSD	aug-cc-pVDZ	0.02	0.05	0.06	4.7	0.06
CC2	aug-cc-pVDZ	0.47	0.47	0.49	58.1	0.11
ADC(2)	aug-cc-pVDZ	0.52	0.52	0.54	62.7	0.14
SCS-ADC(2)	aug-cc-pVDZ	0.15	0.16	0.20	16.1	0.13
SOS-ADC(2)	aug-cc-pVDZ	–0.04	0.11	0.13	13.7	0.13
Exp.	–	0.52	0.54	0.55	68.9	0.20

a CCD­(pCCD) and CCSD­(pCCD) refer to CCD and CCSD methods performed using pCCD-optimized natural orbitals or the pCCD reference determinant. See also Table S9 for other basis sets.

**Table 2:** Statistical Error Metrics (ME, MAE, RMSE, MPE, and SD) of 22 Organic Acceptor Molecules Using Various Methods and Basis Sets, Relative to Experimental Reference Values from Ref^123^ ^a^

Method	Basis Set	Errors w.r.t. experiment (*E*_method_ – *E*_ref_)				
		ME [eV]	MAE [eV]	RMSE [eV]	MPE [%]	SD [eV]
EA-EOM-fpCCD	aug-cc-pVDZ	–0.62	0.65	0.69	58.9	0.30
	cc-pVDZ	–1.00	1.00	1.04	91.6	0.30
	cc-pVTZ	–0.55	0.59	0.61	54.0	0.27
	CBS*	–0.37	0.42	0.45	38.3	0.26
EA-EOM-fpCCSD	aug-cc-pVDZ	–0.67	0.69	0.73	61.9	0.28
	cc-pVDZ	–1.10	1.10	1.14	99.7	0.29
	cc-pVTZ	–0.69	0.71	0.74	64.0	0.28
	CBS*	–0.52	0.56	0.59	49.1	0.29
EA-EOM-fpLCCD	aug-cc-pVDZ	–0.72	0.73	0.77	65.4	0.27
	cc-pVDZ	–1.10	1.10	1.14	100.8	0.29
	cc-pVTZ	–0.70	0.72	0.75	65.0	0.28
	CBS*	–0.53	0.56	0.60	50.1	0.29
EA-EOM-fpLCCSD	aug-cc-pVDZ	–0.84	0.84	0.88	72.1	0.26
	cc-pVDZ	–1.26	1.26	1.29	109.2	0.28
	cc-pVTZ	–0.89	0.89	0.92	81.0	0.24
	CBS*	–0.75	0.75	0.79	68.1	0.26
EA-EOM-CCD(pCCD)	aug-cc-pVDZ	–0.51	0.54	0.59	50.8	0.31
	cc-pVDZ	–0.87	0.88	0.92	82.1	0.29
	cc-pVTZ	–0.44	0.49	0.52	44.9	0.28
	CBS*	–0.26	0.34	0.39	29.8	0.30
EA-EOM-CCSD(pCCD)	aug-cc-pVDZ	–0.55	0.58	0.62	53.8	0.30
	cc-pVDZ	–0.95	0.96	0.99	87.1	0.29
	cc-pVTZ	–0.54	0.58	0.60	52.8	0.28
	CBS*	–0.37	0.42	0.46	38.4	0.28
DIP/IP-EOM-fpCCD	aug-cc-pVDZ	–0.79	0.93	1.20	92.6	0.92
	cc-pVDZ	–0.54	0.58	0.64	51.6	0.35
	cc-pVTZ	–0.03	0.20	0.31	15.3	0.32
	CBS*	0.19	0.24	0.36	15.0	0.31
DIP/IP-EOM-fpCCSD	aug-cc-pVDZ	–0.76	0.87	1.14	87.9	0.87
	cc-pVDZ	–0.63	0.66	0.70	59.0	0.31
	cc-pVTZ	–0.13	0.22	0.30	19.0	0.28
	CBS*	0.09	0.17	0.28	12.2	0.27
DIP/IP-EOM-fpLCCD	aug-cc-pVDZ	–0.73	0.93	1.19	92.2	0.96
	cc-pVDZ	–0.45	0.50	0.56	44.0	0.35
	cc-pVTZ	0.09	0.21	0.32	15.0	0.32
	CBS*	0.31	0.35	0.43	22.6	0.31
DIP/IP-EOM-fpLCCSD	aug-cc-pVDZ	–1.17	1.37	2.09	107.7	1.78
	cc-pVDZ	–0.58	0.99	1.48	72.4	1.40
	cc-pVTZ	–0.24	0.51	0.95	30.0	0.94
	CBS*	–0.09	0.81	1.58	45.6	1.62
DIP/IP-EOM-CCD(pCCD)	aug-cc-pVDZ	–0.81	0.93	1.20	92.8	0.91
	cc-pVDZ	–0.58	0.62	0.68	55.0	0.36
	cc-pVTZ	–0.06	0.22	0.33	17.0	0.33
	CBS*	0.16	0.23	0.35	14.3	0.32
DIP/IP-EOM-CCSD(pCCD)	aug-cc-pVDZ	–0.74	0.84	1.11	85.2	0.84
	cc-pVDZ	–0.68	0.71	0.75	63.4	0.33
	cc-pVTZ	–0.15	0.24	0.30	21.3	0.26
	CBS*	0.08	0.16	0.26	12.2	0.25
Δ-CCSD(T)^121,123^	aug-cc-pVDZ	–0.52	0.54	0.55	48.7	0.20
EA-EOM-CCSD^123^	aug-cc-pVDZ	–0.49	0.53	0.54	48.4	0.23
CC2 ^123^	aug-cc-pVDZ	–0.04	0.18	0.27	15.4	0.28
ADC(2)^123^	aug-cc-pVDZ	0.02	0.18	0.29	13.8	0.29
SCS-ADC(2)^123^	aug-cc-pVDZ	–0.36	0.42	0.45	38.4	0.28
SOS-ADC(2)^123^	aug-cc-pVDZ	–0.55	0.59	0.61	53.0	0.28

a Values marked with * are extrapolated to the complete basis set limit, as described according to eq 18. CCD­(pCCD) and CCSD­(pCCD) refer to CCD and CCSD methods performed using pCCD-optimized natural orbitals or the pCCD reference determinant .

#### 4.1.1 Performance of aug-cc-pVDZ Basis: Comparison to Experiment and Δ-CCSD­(T)

We begin our analysis with the EAs computed using the aug-cc-pVDZ basis set, which includes diffuse functions essential for accurately describing anionic states and electron affinities using the delocalized canonical Hartree–Fock orbitals.^117,125^ Among the theoretical methods listed in Table 2, the best agreement with experiment is obtained for the computationally most expensive Δ-CCSD­(T) approach with SD = 0.20, ME = −0.52, and RMSE = 0.55 eV. To that end, this method is used as a theoretical reference in Table 1. The accuracy of our EA-EOM-fp­(L)­CC methods approaches that of EA-EOM-CCSD, and it is similar to the SCS-ADC(2) and SOS-ADC(2) methods. The best performance in terms of ME and RMSE is obtained for the EA-EOM-fpCCD and EA-EOM-fpCCSD models. The linearized variant of these methods (EA-EOM-fpLCCD and EA-EOM-fpLCCSD) slightly worsens their statistical errors, increasing the ME and RMSE to approximately 0.7–0.9 eV. Compared to experimental EAs, all methods exhibit a systematic underestimation, with average shifts of 0.5–0.7 eV. This underestimation stems from both basis set incompleteness and electron correlation effects not captured by lower CC orders. Nonetheless, the relative ordering of EA values across the molecular test set is well preserved (see Supporting Information for numerical values), indicating that these methods remain suitable for qualitative comparisons or extensive data screening. EA-EOM-CC­(S)­D­(pCCD) methods, which include (single and) double excitations using the pCCD orbital basis (or, equivalently, the pCCD-optimized reference determinant), achieve lower MEs and RMSEs than their frozen-pair counterparts. However, their performance is still worse than the standard EA-EOM-CCSD approach. These observations suggest that splitting the zero- and nonzero-seniority sectors during the optimization has a stronger effect on EAs than the choice of the reference determinant (or the underlying choice of molecular orbitals).

We observe overall better accuracy of our EA-EOM-CC models w.r.t. Δ-CCSD (T) than w.r.t. experiment (cf. compare Tables 2 and 1). Excellent is the performance of EA-EOM-fpCC­(S)­D and EA-EOM-CC­(S)­D­(pCCD) methods with MAEs in the range of 0.08 and 0.25 eV and SDs comparable with a EA-EOM-CCSD using the aug-cc-pVDZ basis set. Yet, the above-mentioned models statistically outperform the CC2 and ADC(2) methods for predicting EAs.

Figure 4 shows the violin plots of our new methods w.r.t. experimental and theoretical reference EAs using the aug-cc-pVDZ basis set. The violin plots allow us to assess the probability distribution of errors using kernel density estimation, and thus visual assessment of the central tendency, spread, and skewness of EA prediction errors. Specifically, the white dot marks the median, and the thick black bar shows the interquartile range (IQR)—the measure of how spread out the middle 50% of the data is. Wider sections of the violin plot indicate higher data density, while narrower sections indicate lower density. It is clear from Figure 4 that all of our EA-EOM-fpCC methods have errors centered around the reference, slightly underestimating experimental values. Their spread is comparable to other theoretical methods, such as ADC or CC2. When compared to the theoretical reference (cf. Figure 4a), the errors from EA-EOM-fpCC and EA-EOM-CC­(pCCD) methods and their median are positioned more in the center of the reference than the ADC or CC2 methods, but with a larger spread.

**Figure 4:**
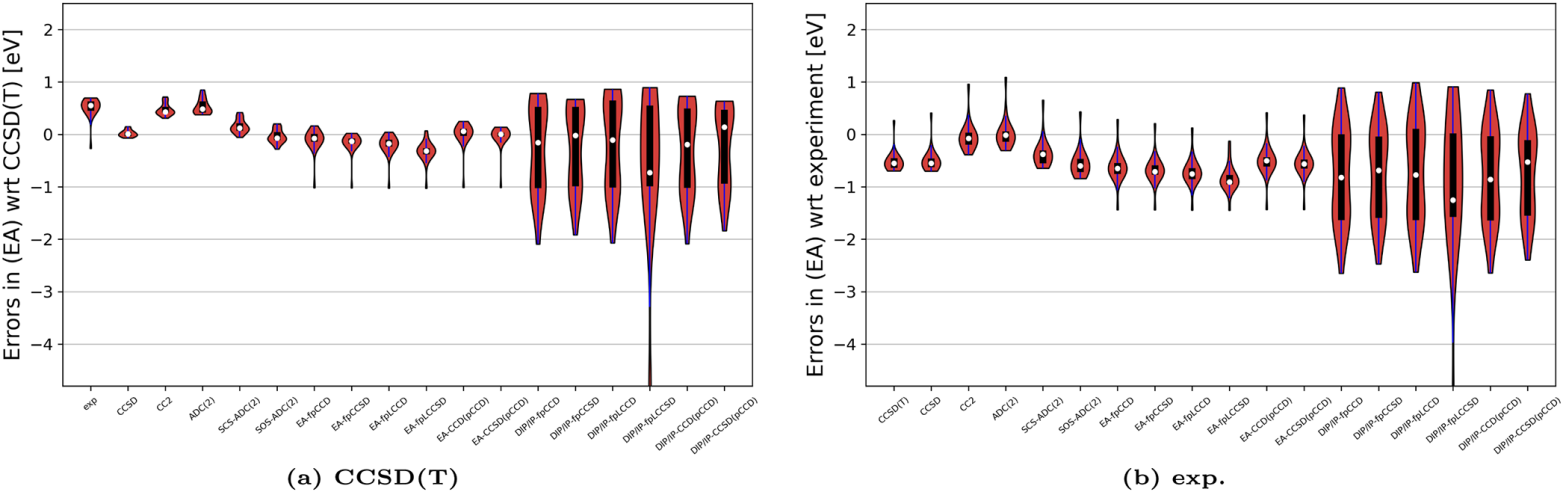
Violin plots illustrating the errors [eV] from selected methods for the test set of organic acceptor molecules. Numerical values are provided in Tables S1–S4. All errors are reported relative to either (a) Δ-CCSD­(T)/aug-cc-pVDZ or (b) experimental reference values, taken from refs 121,123 . Calculations are performed using the aug-cc-pVDZ basis set. For brevity, the EOM prefix is omitted from all EA-EOM-CC and DIP/IP-EOM-CC method labels. The CCD­(pCCD)/CCSD­(pCCD) labels denote conventional EA-EOM-CCD/EA-EOM-CCSD calculations performed using pCCD-optimized (noncanonical) natural orbitals as the reference.

#### 4.1.2 Basis Set and Extrapolation Dependence

To assess the sensitivity of EA predictions to the choice of basis set, we computed them using the cc-pVDZ, cc-pVTZ, and aug-cc-pVDZ basis sets, followed by two-point complete basis set (CBS) extrapolation.^119^ A clear trend emerges: electron affinities improve, becoming less negative and closer to reference values with increasing basis set size. For instance, for EA-EOM-fpCCD, the RMSE drops from 1.14 eV (cc-pVDZ) to 0.74 eV (cc-pVTZ) when referenced against experiment. Compared to experiment, the statistical performance of EA-EOM-fp (L)­CCSD and EA-EOM-CC­(S)­D­(pCCD) methods is similar in cc-pVTZ and aug-cc-pVDZ basis sets (cf. Table 2). In general, adding augmented functions to the basis set (here, going from cc-pVDZ to aug-cc-pVDZ) generally improves EAs, significantly reducing error measures. Furthermore, errors in EAs are similar for the aug-cc-pVDZ and the cc-pVTZ basis set if pCCD-optimized orbitals are used. This observation suggests that the accuracy in EAs can be improved by increasing the basis set size through augmentation or w.r.t. the cardinal number when pCCD-optimized orbitals are employed. CBS extrapolation improves the accuracy further, reducing the statistical errors by approximately 15% compared to cc-pVTZ/aug-cc-pVDZ results for all EA-EOM-fp­(L)­CC and EA-EOM-CC­(S)­D­(pCCD) methods. In the CBS limit, the EA-EOM-fpCCD and EA-EOM-CCD­(pCCD) are the closest to experiment with ME= −0.39 and RMSE= 0.45 eV, and ME= −0.26 and RMSE= 0.39 eV, respectively. The performance of EA-EOM-fpCCSD and EA-EOM-fpLCCD is similar, but the most significant deviation from experiment is observed for the EA-EOM-fpLCCSD approach. We should note that a similar trend between the fp­(L)­CC­(S)­D methods for the accuracy of ground-state dipole moments was observed, with the fpLCCSD approach showing the worst performance.^65^ For reasons of completeness, the convergence of our EA-EOM-CC models w.r.t. basis set size and Δ-CCSD­(T)/aug-cc-pVDZ reference is provided in Table S9 of the Supporting Information.

Figure 5 depicts the violin plots of the CBS extrapolated EAs investigated in this work. All the EA-EOM-fpCC and EA-EOM-CC­(pCCD) methods underestimate the experimental EA values, with EA-EOM-fpLCCSD having the lowest median. The overall error distribution and spread are comparable among these methods. Finally, we should note that a similar behavior for the CBS extrapolation was observed previously.^70^ In particular, the CBS-extrapolated limit of EA-EOM-pCCD was insensitive to diffuse functions, where the aug-cc-pVXZ and conventional cc-pVXZ basis set series yield similar extrapolated EAs.

**Figure 5:**
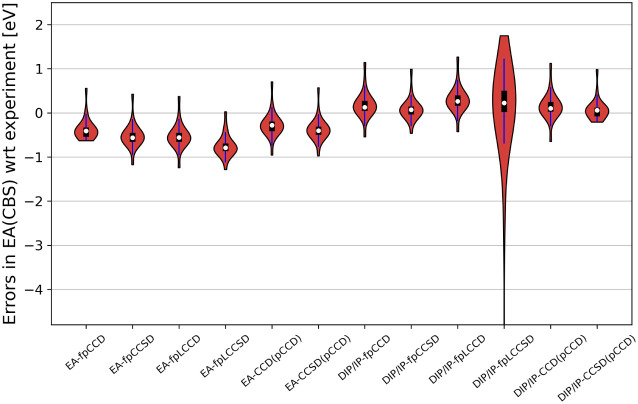
Violin plots illustrating the errors [eV] of electron affinities obtained from both direct EA- and DIP/IP-EOM-CC methods (see eq 15) extrapolated to the basis set limit (DZ–TZ extrapolation). Errors are reported relative to experimental reference data (see Tables S5 and S6 for numerical details). Experimental reference data are taken from ref.^123^ CCD­(pCCD)/CCSD­(pCCD) label conventional EA-EOM-CCD/EA-EOM-CCSD calculation on top of the pCCD reference determinant, that is, using noncanonical pCCD-optimized natural orbitals.

### 4.2 EAs from DIP/IP-EOM-CC Methods

In this subsection, we explore an alternative route for computing EAs using the energy difference between double and single ionization potentials (DIP and IP) of dicationic species (assuming uncharged neutral molecules in EA-EOM-CC calculations).^126–128^ These indirect EAs are calculated as EA = DIP – IP, within the same fpCC reference framework (see also Section 2.5).^50,129–132^

#### 4.2.1 Performance in the aug-cc-pVDZ Basis Set: Comparison to Experiment and CCSD­(T)

First, we examine the aug-cc-pVDZ basis set w.r.t experiment in Table 2. The EAs computed from DIP/IP-EOM-fp­(L)­CC methods perform worse than their EA-EOM-fp­(L)­CC counterparts in this basis set. Still, they correctly reproduce trends among the investigated molecules (see Supporting Information). The best agreement with experiment is achieved for the DIP/IP-EOM-fpCCSD model, which only slightly outperforms its DIP/IP-EOM-fpCCD and DIP/IP-EOM-fpLCCD counterparts. The DIP/IP-EOM-fpLCCSD results exhibit the most significant statistical errors with respect to experiment. The performance of DIP/IP-EOM-CC­(S)­D­(pCCD) methods is similar to their EA-EOM-CC­(S)­D­(pCCD) counterparts.

The middle part of Table 1 compares the DIP/IP-EOM-CC methods to the reference Δ-CCSD­(T) values. We observe slightly larger statistical errors for the DIP/IP-EOM-CC approaches than the corresponding results from direct EA-EOM-CC models. For instance, the MAE = 0.32 eV of the EA-EOM-fpLCCD model increases to MAE = 0.72 eV within the DIP/IP-EOM-fpLCCD approach.

Figure 4 shows the violin plots of the errors obtained by the investigated methods w.r.t. experiment and Δ-CCSD­(T) using the aug-cc-pVDZ basis set. All the DIP/IP-EOM-CC methods have a similar spread of errors, much larger than observed for the EA-EOM-CC models. Furthermore, all investigated DIP/IP-EOM-CC flavors feature a dumbbell-shaped error distribution, highlighting an asymmetric distribution of errors around the median. The worst-performing model is the EOM-fpLCCSD one, with a median value below the reference point of approximately 1 eV.

#### 4.2.2 Basis Set Dependence and CBS with Respect to Experiment

Next, we evaluate the basis set dependence of DIP/IP-EOM-CC-based EAs across the cc-pVDZ, cc-pVTZ, and aug-cc-pVDZ series, and CBS-extrapolated values. Similar to what we observed for the EA-EOM-CC methods, increasing basis set size from cc-pVDZ to cc-pVTZ, and to CBS significantly improves the accuracy of predicted EAs. However, for the DIP/IP-EOM-CC approaches, the improvement toward experimental reference values is more pronounced, and statistical errors reduce more quickly when moving from the cc-pVDZ to cc-pVTZ basis sets. Notably, the CBS extrapolated DIP/IP-EOM-CC EAs are in excellent agreement with experiment, outperforming their EA-EOM-CC variants. The best performance is obtained for the DIP/IP-EOM-fpCCSD and DIP/IP-EOM-CC­(S)­D­(pCCD) methods. However, the presence of augmented functions makes the predicted EAs less accurate compared to experiment, as shown in Table 2. A useful diagnostic for assessing the quality of basis sets in EA computations is the vertical detachment energy (VDE), that is, the energy required to remove an electron in a molecule without relaxing the molecular geometry. While diffuse functions often improve the description of anions in conventional canonical frameworks,^133,134^ we found that with pCCD-optimized orbitals, they instead slow convergence and worsen the accuracy of indirect DIP/IP-based EAs. Thus, reliable predictions can already be achieved without augmented functions when using localized pCCD orbitals.

Furthermore, the aug-cc-pVDZ and CBS results deviate strongly if Δ-CCSD­(T) values are chosen as reference data (see Table 1). The best agreement with the reference theoretical EAs is obtained with the DIP/IP-EOM-fp­(L)­CC methods for the cc-pVDZ basis set.

Relative to Δ-CCSD­(T), the largest finite-basis deviation occurs at the aug-cc-pVDZ level for F_4_benzoquinone in DIP/IP-EOM-fpLCCSD (−5.68 eV), with a similarly large error for dinitrobenzonitrile (−4.93 eV). With respect to experiment, the strongest CBS-level deviation is found for dinitrobenzonitrile in the same (indirect) DIP/IP-EOM-fpLCCSD framework for EA estimations (−6.50 eV), followed by F_4_benzoquinone (−2.33 eV). Furthermore, in the direct EA-EOM fp­(L)­CC variants, the largest CBS-level deviations w.r.t. experiment occur for *dichlone* for EA-EOM-fpLCCSD (with errors up to −1.28 eV). In contrast, the more robust DIP/IP-EOM-CCSD­(pCCD) flavor yields substantially smaller deviations, with maximum CBS errors limited to about 0.98 eV (for TCNQ).

The violin plots (cf. Figure 5) highlight the good performance of DIP/IP-EOM-fp­(L)­CC methods without augmented functions when predicting the CBS extrapolated EAs. Except of DIP/IP-EOM-fpLCCSD, all the investigated models have median centered around the reference point, a very small spread, and IQR, making them very reliable candidates for accurate EA predictions.

#### 4.2.3 Dependence on Single Excitations

Finally, we examine the role of single excitations in DIP/IP-EOM-CC methods. In general, single excitations lower the predicted EAs compared to the doubles-only CC calculations, which results in larger ME values as EAs are consistently underestimated in all EOM-fp­(L)­CC methods (both EA and DIP/IP). Moreover, single excitations (as in DIP/IP-fpCCSD or DIP/IP-CCSD­(pCCD)) significantly reduce both systematic errors and the method spread. The only exception is the DIP/IP-EOM-fpLCCSD approach, where for some of the investigated systems the error in EAs significantly increases. For example, comparing DIP/IP-EOM-fpCCD (MAE ≈ 0.93 eV) and DIP/IP-EOM-fpCCSD (MAE ≈ 0.87 eV) and DIP/IP-EOM-CCD­(pCCD) (MAE ≈ 0.93 eV) and DIP/IP-EOM-CCSD­(pCCD) (MAE ≈ 0.84 eV) using the aug-cc-pVDZ level shows clear improvement. This trend is even more pronounced for CBS-extrapolated values, where the agreement with experiment and the reference Δ-CCSD­(T) is greatly improved with the presence of single excitations (see Figure 5). The influence of single excitation in CBS-extrapolated EAs might lead us to the wrong conclusion that the errors increase for EA-EOM-CC methods, while they decrease for the DIP/IP-EOM-CC recipe. However, the CBS-extrapolated EAs of DIP/IP-EOM-CC-type methods are consistently overestimated. Adding single excitations in the ansatz lowers EAs, and hence reduces the predicted error measures (see Table 2 or Figure 5). Thus, in the CBS limit, DIP/IP-EOM-CC-predicted EAs benefit when *T*_1_ is included in the CC ansatz, while EA-EOM-CC-predicted ones do not. Finally, reasonable results (compared to the experiment) are already obtained for a cc-pVTZ basis set using the DIP/IP-EOM recipe and neglecting single excitations (for instance, DIP/IP-EOM-fpCCD results in ME = −0.28 eV). Notably, when the pCCD orbitals are utilized, a good agreement with reference data is obtained even without augmented functions in the basis set (cf. Tables 1 and 2). Similar observations regarding pCCD orbitals have been made by examining EAs from the modified Koopmans’ theorem^15^ and the simple EA-EOM-pCCD model.^70^

## 5 Conclusions and Outlook

In this work, we derived the working equations for the EA-EOM-fpCCD, EA-EOM-fpCCSD, EA-EOM-fpLCCD, and EA-EOM-fpLCCSD methods and implemented them in the PyBEST software package.^111,112^ All proposed methods (in addition to the conventional EA-EOM-CCD and EA-EOM-CCSD approaches) were systematically benchmarked against a diverse set of 24 organic acceptor molecules using localized pCCD-optimized orbitals in conjunction with the aug-cc-pVDZ, cc-pVDZ, cc-pVTZ basis sets, and CBS-extrapolated EAs. Two sets of reference data were selected, namely, EAs determined from canonical Δ-CCSD­(T) calculations and experimental values. Moreover, we compared the influence of canonical Hartree–Fock orbitals and the natural pCCD-optimized ones on EAs determined from conventional CC methods. We further evaluated the performance of indirect DIP/IP-EOM-CC-based approaches for predicting EAs. In this protocol, EAs are computed as energy differences between double and single ionization potentials of dicationic reference states (*N* + 2 electron states), within the same fpCC framework. Both direct and indirect EA methods significantly improve the accuracy when approaching the CBS extrapolated basis set limit. The smallest statistical errors and spreads centered around the reference point are obtained for the DIP/IP-EOM-fpCCSD method (MAE = 0.22 eV w.r.t. experiment), followed by its DIP/IP-EOM-fpCCD, and DIP/IP-EOM-fpLCCSD variants (cf. Table 2 and Figure 5). EA-EOM-fp­(L)­CCSD methods also provide satisfactory results, approaching the accuracy of the CC2 and ADC methods for the investigated systems. Among the EA-EOM-fp­(L)­CCSD models, the EA-EOM-fpCCD approach was the most accurate across the basis sets tested. The importance of single excitations in the proposed fpCC-based models is negligible when working with the small basis sets, but becomes more pronounced for larger basis sets and CBS values, especially for the DIP/IP-EOM-fpCC methods. Overall, including *T*_1_ in the CC ansatz consistently lowers the predicted EAs, irrespectively of the applied EOM recipe or CC reference state. While EA-EOM-(fp­[L])­CC consistently underestimates EAs, the corresponding DIP/IP-EOM variants tend to overestimate EAs when reaching the CBS limit. Thus, adding single excitations to the DIP/IP-EOM-(fp­[L])­CC ansatz shifts the CBS limit values toward the experimental reference.

Our study demonstrates that when working with the pCCD-optimized orbital basis, there is no need for augmented basis functions to obtain accurate and reliable EAs, contrary to what is genuinely known when working with canonical Hartree–Fock orbitals. Reliable EAs (compared to experimental reference data) were already obtained for the DIP/IP-EOM-fpCCD variant employing the cc-pVTZ basis set, yielding a ME of −0.03 eV. Thus, the introduced pCCD-based approaches have great potential for application in quantum chemical modeling of large molecular systems and EAs prediction, for which standard reliable electronic structure methods are too expensive and/or smaller basis sets are preferred. To that end, these newly developed approaches, combined with the GPU acceleration^58^ possibilities as implemented in the open-source PyBEST software package, represent an up-and-coming alternative for large-scale EA predictions in organic electronics.

## Supplementary Material



## Data Availability

The data underlying this study are available in the published article and its Supporting Information. The released version of the PyBEST code is available on Zenodo at https://zenodo.org/records/10069179 and on PyPI at https://pypi.org/project/pybest/.

## Notes

Working equations for all investigated EA-EOM-fpCC theoretical models, differences between electron affinities from all EA-EOM-fpCC/EA-EOM-CC and DIP/IP-EOM-fpCC or DIP/IP-EOM-CC methods, and reference data for various basis set sizes and molecular orbitals (pCCD and HF ones), additional violin plots illustrating statistical errors, equations used to compute statistical analysis, and EAs obtained from different Cholesky thresholds (pdf); EAs from various EA-EOM-fpCC/EA-EOM-CC models and DIP/IP-EOM-CC energies for cc-pVDZ, cc-pVTZ, aug-cc-pVDZ, and CBS limit (xlsx) (ZIP)
