# Supplementary material for: Electron Affinities from Equation-of-Motion Frozen Pair-Type Coupled Cluster Methods and Their Dependence on Single Excitations, Molecular Orbitals, and Basis Set Sizes
Source: J Chem Theory Comput. 2025 Oct 6;21(20):10315–28. doi: 10.1021/acs.jctc.5c01258 (PMC12573755; doi:10.1021/acs.jctc.5c01258)
Supplement: Supplementary file 1 [file ct5c01258_si_001.zip › si/si.pdf]

Electron Affinities from Equation of Motion Frozen Pair-Type Coupled Cluster Methods and their Dependence on Single Excitations, Molecular Orbitals, and Basis Set Sizes

Saman Behjou,[a] Paweł Tecmer[a] and Katharina Boguslawski,[a*]

[a]*Institute of Physics, Faculty of Physics, Astronomy and Informatics, Nicolaus Copernicus University in Toruń, Grudziądzka 5, 87-100 Toruń, Poland*

*Email: k.boguslawski@fizyka.umk.pl

Supplementary Information

S1 EA-EOM-fpCC and EA-EOM-fpLCC working equations

For $S_z = -\frac{1}{2}$ states, we restrict the operator $\hat{R}^{\text{EA}}(k)$ to 1p (one particle) and 2p1h (two particle one hole) terms, and the following spin blocks

$$\hat{R}^{\text{EA}}_{S_z=-\frac{1}{2}}(k) = \hat{R}^{\alpha}_{1p}(k) + \hat{R}^{\alpha\alpha\alpha}_{2p1h}(k) + \hat{R}^{\alpha\beta\beta}_{2p1h}(k), \tag{1}$$

with

$$\hat{R}^{\alpha}_{1p}(k) = \sum_a r^a(k)\hat{a}^\dagger, \tag{2}$$

$$\hat{R}^{\alpha\alpha\alpha}_{2p1h}(k) = \frac{1}{2}\sum_{abj} r^{ab}_j(k) a^\dagger b^\dagger j, \tag{3}$$

and

$$\hat{R}^{\alpha\beta\beta}_{2p1h}(k) = \sum_{a\bar{b}\bar{j}} r^{a\bar{b}}_{\bar{j}}(k) a^\dagger \bar{b}^\dagger \bar{j}. \tag{4}$$

The $\hat{R}$ amplitudes satisfy the symmetry relation $r^{ab}_j = -r^{ba}_j$. The configurational subspace during diagonalization is spanned by $|\Phi^a\rangle, \left|\Phi^{ab}_j\right\rangle, \left|\Phi^{a\bar{b}}_{\bar{j}}\right\rangle$. The working equations for the various EA-EOM flavors (for each $\hat{R}$ block) mentioned here read as follows (all integrals are represented in the restricted orbital basis, that is, $\langle pq|rs\rangle = \langle p\bar{q}|r\bar{s}\rangle = \langle \bar{p}q|\bar{r}s\rangle = \langle \bar{p}\bar{q}|\bar{r}\bar{s}\rangle$; summation over repeated indices is implied)

$$\begin{aligned}(\bar{H}\hat{R})^a = {} & I_{ad}r^d + \left(\frac{1}{2}\langle ak||dc\rangle - \frac{1}{2}\langle mk||dc\rangle\, t^a_m\right) r^{dc}_k \\ & + \left(\left\langle a\bar{k}\middle|d\bar{c}\right\rangle - \left\langle m\bar{k}\middle|d\bar{c}\right\rangle t^a_m\right) r^{d\bar{c}}_{\bar{k}} + I_{ck}r^{ac}_k + I_{ck}r^{a\bar{c}}_{\bar{k}},\end{aligned} \tag{5}$$

$$\begin{aligned}(\bar{H}\hat{R})^{ab}_j = \mathcal{P}^-(ab)\Bigg[& I_{abjd}r^d + I_{bd}r^{ad}_j + \frac{1}{2}I_{jm}r^{ab}_m + I_{abcd}r^{cd}_j + I_{jbkc}r^{ac}_k + I_{jbKC}r^{a\bar{c}}_{\bar{k}} \\ & - \frac{1}{4}\langle mk\|dc\rangle\left(t^{ab}_{mj} - t^{ab}_{jm}\right) r^{dc}_k - \frac{1}{2}\langle m\bar{k}|d\bar{c}\rangle\left(t^{ab}_{mj} - t^{ab}_{jm}\right) r^{d\bar{c}}_{\bar{k}}, \Bigg]\end{aligned} \tag{6}$$

with the permutation operation defined as

$$\mathcal{P}^-(ab) = 1 - \hat{P}_{ab}, \tag{7}$$

and

$$\begin{aligned}(\bar{H}\hat{R})^{a\bar{b}}_{\bar{j}} = {} & I_{aBJd}r^d + I_{bd}r^{a\bar{d}}_{\bar{j}} + I_{ad}r^{d\bar{b}}_{\bar{j}} + I_{jm}r^{a\bar{b}}_{\bar{m}} + I_{aBcD}r^{c\bar{d}}_{\bar{j}} + I_{jbKC}r^{ac}_k + I_{jbkc}r^{a\bar{c}}_{\bar{k}} + I_{JaKc}r^{c\bar{b}}_{\bar{k}} \\ & - \frac{1}{2}\langle mk\|dc\rangle t^{ab}_{mj}r^{dc}_k - \langle m\bar{k}|d\bar{c}\rangle t^{ab}_{mj}r^{d\bar{c}}_{\bar{k}}\end{aligned} \tag{8}$$

In the above equations, we used the spin-free amplitudes

$$t_i^a = t_{i_\alpha}^{a_\alpha} = t_{i_\beta}^{a_\beta}, \tag{9}$$

$$t_{ij}^{ab} = t_{i_\alpha j_\beta}^{a_\alpha b_\beta}, \tag{10}$$

and

$$t_{ij}^{ab} - t_{ij}^{ba} = t_{i_\alpha j_\alpha}^{a_\alpha b_\alpha}. \tag{11}$$

The intermediates (using spin-free amplitudes) for a fpCCSD reference function read as follows

$$\begin{aligned} I_{ad} = {} & f_{ad} - f_{md} t_m^a - \frac{1}{2} \langle mk||dc\rangle (t_{mk}^{ac} - t_{km}^{ac}) - \langle m\bar{k}|d\bar{c}\rangle t_{mk}^{ac} \\ & + \langle ka||cd\rangle t_k^c - \langle mk||dc\rangle t_m^a t_k^c - \langle m\bar{k}|d\bar{c}\rangle t_m^a t_k^c + \langle \bar{k}a|\bar{c}d\rangle t_k^c \end{aligned} \tag{12}$$

$$I_{bd} = I_{ad}(b,d) \tag{13}$$

$$I_{ck} = f_{kc} + \langle km||cd\rangle t_m^d + \langle k\bar{m}|c\bar{d}\rangle t_m^d \tag{14}$$

$$\begin{aligned} I_{abjd} = {} & [\frac{1}{2} \langle ab||dj\rangle - \langle am||dj\rangle t_m^b + \frac{1}{2} \langle ab||dc\rangle t_j^c - \frac{1}{2} f_{kd}(t_{kj}^{ab} - t_{jk}^{ab}) \\ & + \langle ak||dc\rangle (t_{jk}^{bc} - t_{jk}^{cb}) + \langle a\bar{k}|d\bar{c}\rangle t_{jk}^{bc} + \frac{1}{4} \langle mk||dj\rangle (t_{mk}^{ab} - t_{km}^{ab}) \\ & - \langle mb||dc\rangle t_j^c t_m^a + \frac{1}{2} \langle mk||dj\rangle t_m^a t_k^b - \langle mk||dc\rangle t_m^a (t_{jk}^{bc} - t_{kj}^{bc}) \\ & - \langle m\bar{k}|d\bar{c}\rangle t_m^a t_{jk}^{bc} - \frac{1}{2} \langle mk||dc\rangle t_k^c (t_{mj}^{ab} - t_{jm}^{ab}) \\ & - \frac{1}{2} \langle m\bar{k}|d\bar{c}\rangle t_k^c (t_{mj}^{ab} - t_{jm}^{ab}) + \frac{1}{2} \langle mk||dc\rangle t_j^c (t_{mk}^{ab} - t_{km}^{ab}) \\ & + \frac{1}{2} \langle mk||dc\rangle t_k^b t_m^a t_j^c] \end{aligned} \tag{15}$$

$$\begin{aligned} I_{aBJd} = {} & [\, \langle a\bar{b}|d\bar{j}\rangle - \langle a\bar{m}|d\bar{j}\rangle t_m^b + \langle m\bar{b}|d\bar{j}\rangle t_m^a + \langle a\bar{b}|d\bar{c}\rangle t_j^c \\ & - f_{kd} t_{kj}^{ab} + \langle ak||dc\rangle t_{jk}^{bc} + \langle ak|dc\rangle (t_{jk}^{bc} - t_{kj}^{bc}) \\ & - \langle m\bar{b}|d\bar{c}\rangle t_{mj}^{ac} + \langle m\bar{k}|d\bar{j}\rangle t_{mk}^{ab} - \langle m\bar{b}|d\bar{c}\rangle t_j^c t_m^a \\ & - \langle a\bar{m}|d\bar{c}\rangle t_j^c t_m^b + \langle m\bar{k}|d\bar{j}\rangle t_m^a t_k^b - \langle mk||dc\rangle t_m^a t_{jk}^{bc} \\ & - \langle m\bar{k}|d\bar{c}\rangle t_m^a (t_{jk}^{bc} - t_{kj}^{bc}) + \langle m\bar{k}|d\bar{c}\rangle t_k^b t_{mj}^{ac} - \langle mk||dc\rangle t_k^c t_{mj}^{ab} \\ & - \langle m\bar{k}|d\bar{c}\rangle t_k^c t_{mj}^{ab} + \langle m\bar{k}|d\bar{c}\rangle t_j^c t_{mk}^{ab} + \langle m\bar{k}|d\bar{c}\rangle t_k^b t_m^a t_j^c] \end{aligned} \tag{16}$$

$$\begin{aligned} I_{jm} = {} & -f_{mj} - \frac{1}{2} \langle mk||dc\rangle (t_{jk}^{dc} - t_{kj}^{dc}) - \langle m\bar{k}|d\bar{c}\rangle t_{jk}^{dc} - \langle mk||dc\rangle t_j^d t_k^c \\ & - \langle m\bar{k}|d\bar{c}\rangle t_j^d t_k^c - f_{mc} t_j^c - \langle mk||jc\rangle t_k^c - \langle m\bar{k}|j\bar{c}\rangle t_k^c \end{aligned} \tag{17}$$

$$I_{abcd} = \frac{1}{4}\left\langle ab||cd\right\rangle + \frac{1}{8}\left\langle km||dc\right\rangle (t^{ab}_{mk} - t^{ab}_{km}) + \frac{1}{4}\left\langle km||dc\right\rangle t^a_k t^b_m - \frac{1}{2}\left\langle mb||cd\right\rangle t^a_m \tag{18}$$

$$I_{aBCd} = \left\langle a\bar{b}\middle|c\bar{d}\right\rangle + \left\langle k\bar{m}\middle|c\bar{d}\right\rangle t^{ab}_{km} + \left\langle m\bar{k}\middle|c\bar{d}\right\rangle t^a_m t^b_k - \left\langle m\bar{b}\middle|c\bar{d}\right\rangle t^a_m - \left\langle a\bar{m}\middle|c\bar{d}\right\rangle t^b_m \tag{19}$$

$$I_{jbkc} = \left\langle kb||cj\right\rangle + \left\langle kl||cd\right\rangle (t^{bd}_{jl} - t^{bd}_{lj}) + \left\langle kl|cd\right\rangle t^{bd}_{jl} - \left\langle km||cd\right\rangle t^d_j t^b_m + \left\langle kb||cd\right\rangle t^d_j - \left\langle km||cj\right\rangle t^b_m \tag{20}$$

$$I_{jbKC} = \left\langle \bar{k}b\middle|\bar{c}j\right\rangle + \left\langle \bar{k}l\middle|\bar{c}d\right\rangle (t^{bd}_{jl} - t^{bd}_{lj}) + \left\langle kl||cd\right\rangle t^{bd}_{jl} - \left\langle \bar{k}m\middle|\bar{c}d\right\rangle t^d_j t^b_m + \left\langle \bar{k}b\middle|\bar{c}d\right\rangle t^d_j - \left\langle \bar{k}m\middle|\bar{c}j\right\rangle t^b_m \tag{21}$$

$$I_{JaKc} = -\left\langle \bar{k}a\middle|\bar{j}c\right\rangle + \left\langle \bar{k}l\middle|\bar{d}c\right\rangle t^{da}_{jl} + \left\langle \bar{k}m\middle|\bar{d}c\right\rangle t^d_j t^a_m - \left\langle a\bar{k}\middle|c\bar{d}\right\rangle t^d_j + \left\langle m\bar{k}\middle|c\bar{j}\right\rangle t^a_m \tag{22}$$

Note that

$$I_{jbkc} = I_{jbKC} + I_{Jakc}(j, b, k, c) \tag{23}$$

Furthermore, we use the shorthand notation

$$I_{pq} = I_{pq}(p, q) \quad \text{and} \quad I_{pqrs} = I_{pqrs}(p, q, r, s) \tag{24}$$

where capital letters ($I, J, \ldots$ or $A, B, \ldots$) imply β spin indices and are used to distinguish intermediates with similar indices, but belonging to different spin blocks.

For the linearized version of fpCCSD, that is, EA-EOM-fpLCCSD, we approximate the disconnected $\hat{T}_1\hat{T}_2$ term with $\hat{T}_1\hat{T}_\mathrm{p}$, while all remaining disconnected terms are neglected. We also store both pair and broken-pair amplitudes in the $\hat{T}_2$ tensor, that is, $\hat{T}_2 = \hat{T}_2' + \hat{T}_\mathrm{p}$. Thus, we obtain the following modified set of intermediates (using the spin-free amplitudes and integrals represented in the spatial orbital basis)

$$I^{\text{fpLCCSD}}_{ad} = f_{ad} - f_{md} t^a_m - \frac{1}{2}\left\langle mk||dc\right\rangle (t^{ac}_{mk} - t^{ac}_{km}) - \left\langle m\bar{k}\middle|d\bar{c}\right\rangle t^{ac}_{mk} + \left\langle ka||cd\right\rangle t^c_k + \left\langle \bar{k}a\middle|\bar{c}d\right\rangle t^c_k \tag{25}$$

$$I^{\text{fpLCCSD}}_{bd} = I^{\text{fpLCCSD}}_{ad}(b, d) \tag{26}$$

$$I^{\text{fpLCCSD}}_{ck} = I_{ck} \tag{27}$$

$$\begin{aligned} I^{\text{fpLCCSD}}_{abjd} = \Bigg[& \frac{1}{2}\left\langle ab||dj\right\rangle - \left\langle am||dj\right\rangle t^b_m + \frac{1}{2}\left\langle ab||dc\right\rangle t^c_j - \frac{1}{2} f_{kd}(t^{ab}_{kj} - t^{ab}_{jk}) \\ & + \left\langle ak||dc\right\rangle (t^{bc}_{jk} - t^{cb}_{jk}) + \left\langle a\bar{k}\middle|d\bar{c}\right\rangle t^{bc}_{jk} + \frac{1}{4}\left\langle mk||dj\right\rangle (t^{ab}_{mk} - t^{ab}_{km}) \\ & - \left\langle m\bar{j}\middle|d\bar{b}\right\rangle t^a_m t^{b\bar{b}}_{j\bar{j}} \Bigg] \end{aligned} \tag{28}$$

$$I_{aBJd}^{\text{fpLCCSD}} = \Bigg[\langle a\bar{b}|d\bar{j}\rangle - \langle a\bar{m}|d\bar{j}\rangle t_m^b + \langle m\bar{b}|d\bar{j}\rangle t_m^a + \langle a\bar{b}|d\bar{c}\rangle t_j^c - f_{kd}t_{kj}^{ab}$$
$$+ \langle ak||dc\rangle t_{jk}^{bc} + \langle ak|dc\rangle (t_{jk}^{bc} - t_{kj}^{bc}) - \langle m\bar{b}|d\bar{c}\rangle t_{mj}^{ac} + \langle m\bar{k}|d\bar{j}\rangle t_{mk}^{ab}$$
$$- \langle mj||db\rangle t_m^a t_{j\bar{j}}^{b\bar{b}} + \langle j\bar{k}|d\bar{a}\rangle t_k^b t_{j\bar{j}}^{a\bar{a}}$$
$$+ \delta_{ab}\Big(- \langle jk||dc\rangle t_k^c t_{j\bar{j}}^{a\bar{a}} - \langle j\bar{k}|d\bar{c}\rangle t_k^c t_{j\bar{j}}^{a\bar{a}} + \langle m\bar{m}|d\bar{c}\rangle t_j^c t_{m\bar{m}}^{a\bar{a}} \Big)\Bigg] \tag{29}$$

$$I_{jm}^{\text{fpLCCSD}} = -f_{mj} - \frac{1}{2}\langle mk||dc\rangle (t_{jk}^{dc} - t_{kj}^{dc}) - \langle m\bar{k}|d\bar{c}\rangle t_{jk}^{dc} - f_{mc}t_j^c - \langle mk||jc\rangle t_k^c - \langle m\bar{k}|j\bar{c}\rangle t_k^c \tag{30}$$

$$I_{abcd}^{\text{fpLCCSD}} = \frac{1}{4}\langle ab||cd\rangle + \frac{1}{8}\langle km||dc\rangle (t_{mk}^{ab} - t_{km}^{ab}) - \frac{1}{2}\langle mb||cd\rangle t_m^a \tag{31}$$

$$I_{aBCd}^{\text{fpLCCSD}} = \langle a\bar{b}|c\bar{d}\rangle + \langle k\bar{m}|c\bar{d}\rangle t_{km}^{ab} - \langle m\bar{b}|c\bar{d}\rangle t_m^a - \langle a\bar{m}|c\bar{d}\rangle t_m^b \tag{32}$$

$$I_{jbkc}^{\text{fpLCCSD}} = \langle kb||cj\rangle + \langle kl||cd\rangle (t_{jl}^{bd} - t_{lj}^{bd}) + \langle kl|cd\rangle t_{jl}^{bd} + \langle kb||cd\rangle t_j^d - \langle km||cj\rangle t_m^b \tag{33}$$

$$I_{jbKC}^{\text{fpLCCSD}} = \langle \bar{k}b|\bar{c}j\rangle + \langle \bar{k}l|\bar{c}d\rangle (t_{jl}^{bd} - t_{lj}^{bd}) + \langle kl||cd\rangle t_{jl}^{bd} + \langle \bar{k}b|\bar{c}d\rangle t_j^d - \langle \bar{k}m|\bar{c}j\rangle t_m^b \tag{34}$$

$$I_{JaKc}^{\text{fpLCCSD}} = - \langle \bar{k}a|\bar{j}c\rangle + \langle \bar{k}l|\bar{d}c\rangle t_{jl}^{da} - \langle a\bar{k}|c\bar{d}\rangle t_j^d + \langle m\bar{k}|c\bar{j}\rangle t_m^a \tag{35}$$

To obtain the working equations for EA-EOM-fpCCD and EA-EOM-fpLCCD, all T_1 terms in the above equations are to be deleted.

S2 Electron Affinities from EA-EOM-CC and DIP/IP-EOM-CC

Below we collect all energy differences in electron affinities (EAs) with respect to Δ-CCSD(T) or experimental reference data. All EAs are displayed in eV and computed using various pCCD-based theoretical methods and three different basis sets for 24 acceptor molecules. The experimental values are taken from Ref. 1, while theoretical reference data are adopted from the supplementary material of Ref. 2. An alternative approach to describing EAs relies on approximating them via ionization processes using the IP and DIP variants of EOM-CC methods. Having access to the ionization energies E_{IP} and double-ionization energies E_{DIP}, the electron affinity can be estimated as

$$E_{\text{EA}} = E_{\text{DIP}} - E_{\text{IP}} \tag{36}$$

Below, we compare computed EAs against Δ-CCSD(T) results obtained using the aug-cc-pVDZ basis set. We also drop the EA-EOM or DIP-/IP-EOM prefix for the sake of brevity. However, all CC results are obtained using one of the EOM formalisms mentioned above. CCD(pCCD) and

CCSD(pCCD) refer to CCD and CCSD methods performed using pCCD-optimized natural orbitals or the pCCD reference determinant.

The performance of various methods is assessed using the following statistical metrics: Mean Error (ME), Mean Absolute Error (MAE), Root-Mean-Square Error (RMSE), Mean Percentage Error (MPE), and Standard Deviation (SD), defined as

$$\mathrm{ME} = \sum_{i}^{N} \frac{E_i^{\mathrm{method}} - E_i^{\mathrm{ref}}}{N}, \tag{37}$$

$$\mathrm{MAE} = \sum_{i}^{N} \frac{|E_i^{\mathrm{method}} - E_i^{\mathrm{ref}}|}{N}, \tag{38}$$

$$\mathrm{RMSE} = \sqrt{\sum_{i}^{N} \frac{(E_i^{\mathrm{method}} - E_i^{\mathrm{ref}})^2}{N}}, \tag{39}$$

$$\mathrm{MPE} = \frac{1}{N} \sum_{i}^{N} \frac{|E_i^{\mathrm{method}} - E_i^{\mathrm{ref}}|}{E_i^{\mathrm{ref}}} \times 100, \tag{40}$$

$$\mathrm{SD} = \sqrt{\frac{\sum_i^N \left(E_i^{\mathrm{ME}} - \overline{E^{\mathrm{ME}}}\right)^2}{N}}, \tag{41}$$

where method refers to the chosen EA- or DIP/IP-EOM-CC variant to determine EAs, while ref labels the Δ-CCSD(T) or experimental reference values. All sums run over the molecular test set of $N = 24$ organic acceptor molecules in case of Δ-CCSD(T) reference data (aug-cc-pVDZ), while only $N = 22$ data points are available for the experimental reference data (see also Supplementary Information).

Table S1: Energy differences in EAs for various pCCD-based EA-EOM flavors [eV] for the set of 22 organic acceptor molecules using various basis sets. The experimental reference values are taken from Ref. 1.

**Molecule**	**Basis Set**	**EXP**	$\Delta E_{\mathrm{EA}} = E_{\mathrm{method}} - E_{\mathrm{ref}}$					
			fpCCD	fpCCSD	fpLCCD	fpLCCSD	CCD(pCCD)	CCSD(pCCD)
acridine	aug-cc-pVDZ		-1.436	-1.438	-1.443	-1.445	-1.432	-1.432
	cc-pVDZ	0.90	-0.960	-0.981	-1.093	-1.175	-0.856	-0.856
	cc-pVTZ		-0.566	-0.614	-0.716	-0.849	-0.454	-0.480
anthracene	aug-cc-pVDZ		-0.570	-0.567	-0.698	-0.770	-0.469	-0.441
	cc-pVDZ	0.53	-0.912	-0.939	-1.042	-1.136	-0.808	-0.810
	cc-pVTZ		-0.541	-0.593	-0.685	-0.934	-0.427	-0.455
azulene	aug-cc-pVDZ		-0.468	-0.524	-0.589	-0.361	-0.376	-0.425
	cc-pVDZ	0.80	-0.856	-0.942	-0.980	-0.761	-0.760	-0.839
	cc-pVTZ		-0.474	-0.588	-0.612	-0.489	-0.369	-0.476
benzonitrile	aug-cc-pVDZ		-0.681	-0.684	-0.686	-0.692	-0.630	-0.677
	cc-pVDZ	0.26	-1.176	-1.277	-1.270	-1.189	-1.097	-1.097
	cc-pVTZ		-0.719	-0.839	-0.826	-1.040	-0.631	-0.743
benzoquinone	aug-cc-pVDZ		-0.766	-0.755	-0.854	-0.884	-0.605	-0.584
	cc-pVDZ	1.85	-1.221	-1.264	-1.307	-1.393	-1.055	-1.089
	cc-pVTZ		-0.744	-0.801	-0.844	-0.947	-0.567	-0.614
dichlone	aug-cc-pVDZ		-0.647	-0.702	-0.752	-0.949	-0.495	-0.549
	cc-pVDZ	2.21	-1.021	-1.134	-1.123	-1.366	-0.865	-0.979
	cc-pVTZ		-0.609	-1.161	-1.204	-1.036	-0.927	-0.974

Continued on next page

Molecule	Basis Set	EXP	$\Delta E_{\mathbf{EA}} = E_{\mathbf{method}} - E_{\mathbf{ref}}$					
			fpCCD	fpCCSD	fpLCCD	fpLCCSD	CCD(pCCD)	CCSD(pCCD)
fumaronitrile	aug-cc-pVDZ		-0.574	-0.631	-0.640	-0.723	-0.448	-0.494
	cc-pVDZ	1.25	-1.031	-1.129	-1.097	-1.223	-0.900	-0.987
	cc-pVTZ		-0.543	-0.651	-0.621	-0.761	-0.402	-0.497
maleic anhydride	aug-cc-pVDZ		-0.798	-0.835	-0.858	-0.927	-0.658	-0.682
	cc-pVDZ	1.44	-1.251	-1.350	-1.309	-1.446	-1.106	-1.190
	cc-pVTZ		-0.779	-0.881	-0.850	-0.991	-0.625	-0.713
mDCNB	aug-cc-pVDZ		-0.501	-0.583	-0.601	-0.774	-0.422	-0.492
	cc-pVDZ	0.91	-0.916	-1.035	-1.016	-1.228	-0.836	-0.942
	cc-pVTZ		-0.468	-0.602	-0.583	-0.830	-0.380	-0.501
napthalenedione	aug-cc-pVDZ		-0.747	-0.767	-0.850	-0.988	-0.600	-0.617
	cc-pVDZ	1.81	-1.162	-1.231	-1.264	-1.441	-1.011	-1.079
	cc-pVTZ		-0.786	-0.835	-0.899	-0.974	-0.624	-0.660
nitrobenzene	aug-cc-pVDZ		-0.802	-0.836	-0.900	-1.011	-0.665	-0.704
	cc-pVDZ	1.00	-1.478	-1.562	-1.573	-1.712	-1.288	-1.351
	cc-pVTZ		-0.869	-0.987	-0.985	-1.204	-0.712	-0.834
nitrobenzonitrile	aug-cc-pVDZ		-0.653	-0.719	-0.765	-0.933	-0.526	-0.584
	cc-pVDZ	1.69	-1.201	-1.314	-1.310	-1.499	-1.040	-1.123
	cc-pVTZ		-0.647	-0.788	-0.776	-1.048	-0.504	-0.635
phenazine	aug-cc-pVDZ		-0.610	-0.601	-0.757	-0.956	-0.500	-0.471
	cc-pVDZ	1.31	-0.985	-1.015	-1.131	-1.329	-0.873	-0.881
	cc-pVTZ		-0.563	-0.620	-0.728	-1.097	-0.440	-0.477
phthalic anhydride	aug-cc-pVDZ		-0.659	-0.731	-0.757	-0.933	-0.556	-0.621
	cc-pVDZ	1.25	-1.065	-1.196	-1.161	-1.397	-0.961	-1.085
	cc-pVTZ		-0.621	-0.760	-0.732	-1.004	-0.509	-0.642
phthalimide	aug-cc-pVDZ		-0.755	-0.782	-0.847	-0.935	-0.648	-0.659
	cc-pVDZ	1.02	-1.214	-1.296	-1.304	-1.447	-1.104	-1.168
	cc-pVTZ		-0.743	-0.837	-0.849	-1.015	-0.625	-0.703
TCNE	aug-cc-pVDZ		-0.278	-0.405	-0.383	-0.583	-0.153	-0.262
	cc-pVDZ	3.16	-0.596	-0.766	-0.698	-0.940	-0.473	-0.624
	cc-pVTZ		-0.158	-0.332	-0.276	-0.536	-0.023	-0.178
Cl_4benzoquinone	aug-cc-pVDZ		-0.665	-0.723	-0.760	-0.883	-0.497	-0.548
	cc-pVDZ	2.78	-1.017	-1.149	-1.109	-1.310	-0.847	-0.973
	cc-pVTZ		-0.626	-0.755	-0.737	-0.941	-0.446	-0.565
Cl_4isobenzofuranedione	aug-cc-pVDZ		-0.537	-0.636	-0.641	-0.825	-0.428	-0.508
	cc-pVDZ	1.96	-0.798	-0.967	-0.912	-1.153	-0.731	-0.879
	cc-pVTZ		-0.465	-0.632	-0.584	-0.856	-0.350	-0.496
F_4benzenedicarbonitrile	aug-cc-pVDZ		-0.392	-0.535	-0.513	-0.813	-0.309	-0.437
	cc-pVDZ	1.89	-0.710	-0.927	-0.827	-1.194	-0.630	-0.832
	cc-pVTZ		-0.296	-0.498	-0.431	-0.837	-0.206	-0.394
F_4benzoquinone	aug-cc-pVDZ		-0.778	-0.840	-0.874	-1.000	-0.611	-0.656
	cc-pVDZ	2.70	-1.201	-1.351	-1.297	-1.520	-1.031	-1.163
	cc-pVTZ		-0.760	-0.891	-0.871	-1.077	-0.579	-0.695
dinitrobenzonitrile	aug-cc-pVDZ		-0.620	-0.719	-0.726	-0.944	-0.490	-0.577
	cc-pVDZ	2.16	-1.181	-1.327	-1.284	-1.512	-1.012	-1.113
	cc-pVTZ		-0.610	-0.787	-0.732	-1.060	-0.464	-0.624
TCNQ	aug-cc-pVDZ		0.286	0.207	0.125	-0.126	0.415	0.371
	cc-pVDZ	2.80	0.008	-0.113	-0.154	-0.442	0.135	0.135
	cc-pVTZ		0.394	0.265	0.217	-0.113	0.535	0.441

Table S2: Energy differences in EAs for various pCCD-based EA-EOM flavors [eV] for the set of 24 organic acceptor molecules using various basis sets. The Δ-CCSD(T) reference values are taken from Refs. 1,3 and determined for the aug-cc-pVDZ basis set only.

Molecule	Basis Set	CCSD(T)	$\Delta E_{\mathbf{EA}} = E_{\mathbf{method}} - E_{\mathbf{ref}}$					
			fpCCD	fpCCSD	fpLCCD	fpLCCSD	CCD(pCCD)	CCSD(pCCD)
acridine	aug-cc-pVDZ	0.48	-1.016	-1.018	-1.023	-1.025	-1.012	-1.012
	cc-pVDZ		-0.540	-0.561	-0.673	-0.755	-0.436	-0.436
	cc-pVTZ		-0.146	-0.194	-0.296	-0.429	-0.034	-0.060
anthracene	aug-cc-pVDZ	0.141	-0.181	-0.178	-0.309	-0.381	-0.080	-0.052
	cc-pVDZ		-0.523	-0.550	-0.653	-0.747	-0.419	-0.421
	cc-pVTZ		-0.152	-0.204	-0.296	-0.545	-0.038	-0.066

Continued on next page

Molecule	Basis Set	CCSD(T)	$\Delta E_{EA} = E_{method} - E_{ref}$					
			fpCCD	fpCCSD	fpLCCD	fpLCCSD	CCD(pCCD)	CCSD(pCCD)
azulene	aug-cc-pVDZ	0.369	-0.037	-0.093	-0.158	0.070	0.055	0.006
	cc-pVDZ		-0.425	-0.511	-0.549	-0.330	-0.329	-0.408
	cc-pVTZ		-0.043	-0.157	-0.181	-0.058	0.062	-0.045
benzonitrile	aug-cc-pVDZ	-0.431	0.010	0.007	0.005	-0.001	0.061	0.014
	cc-pVDZ		-0.485	-0.586	-0.579	-0.498	-0.406	-0.406
	cc-pVTZ		-0.028	-0.148	-0.135	-0.349	0.060	-0.052
benzoquinone	aug-cc-pVDZ	1.323	-0.239	-0.228	-0.327	-0.357	-0.078	-0.057
	cc-pVDZ		-0.694	-0.737	-0.780	-0.866	-0.528	-0.562
	cc-pVTZ		-0.217	-0.274	-0.317	-0.420	-0.040	-0.087
bodipy	aug-cc-pVDZ	1.472	-0.132	-0.103	-0.259	-0.116	-0.081	-0.038
	cc-pVDZ		-0.397	-0.407	-0.523	-0.351	-0.357	-0.354
	cc-pVTZ		-0.053	-0.079	-0.198	-0.134	-0.012	-0.027
Cl_4benzoquinone	aug-cc-pVDZ	2.224	-0.109	-0.167	-0.204	-0.327	0.059	0.008
	cc-pVDZ		-0.461	-0.593	-0.553	-0.754	-0.291	-0.417
	cc-pVTZ		-0.070	-0.199	-0.181	-0.385	0.110	-0.009
Cl_4isobenzofuranedione	aug-cc-pVDZ	1.411	0.012	-0.087	-0.092	-0.276	0.121	0.041
	cc-pVDZ		-0.249	-0.418	-0.363	-0.604	-0.182	-0.330
	cc-pVTZ		0.084	-0.083	-0.035	-0.308	0.199	0.053
dichlone	aug-cc-pVDZ	1.686	-0.123	-0.178	-0.228	-0.425	0.029	-0.025
	cc-pVDZ		-0.497	-0.610	-0.599	-0.842	-0.341	-0.455
	cc-pVTZ		-0.085	-0.637	-0.680	-0.783	-0.403	-0.450
dinitrobenzonitrile	aug-cc-pVDZ	1.521	0.019	-0.080	-0.087	-0.305	0.149	0.062
	cc-pVDZ		-0.542	-0.688	-0.645	-0.873	-0.373	-0.474
	cc-pVTZ		0.029	-0.148	-0.093	-0.421	0.175	0.015
F_4benzenedicarbonitrile	aug-cc-pVDZ	1.334	0.164	0.021	0.043	-0.257	0.247	0.119
	cc-pVDZ		-0.154	-0.371	-0.271	-0.638	-0.074	-0.276
	cc-pVTZ		0.260	0.058	0.125	-0.281	0.350	0.162
F_4benzoquinone	aug-cc-pVDZ	2.015	-0.093	-0.155	-0.189	-0.315	0.074	0.029
	cc-pVDZ		-0.516	-0.666	-0.612	-0.835	-0.346	-0.478
	cc-pVTZ		-0.075	-0.206	-0.186	-0.392	0.106	-0.010
fumaronitrile	aug-cc-pVDZ	0.728	-0.052	-0.109	-0.118	-0.201	0.074	0.028
	cc-pVDZ		-0.509	-0.607	-0.575	-0.701	-0.378	-0.465
	cc-pVTZ		-0.021	-0.129	-0.099	-0.239	0.120	0.025
maleic anhydride	aug-cc-pVDZ	0.773	-0.131	-0.168	-0.191	-0.260	0.009	-0.015
	cc-pVDZ		-0.584	-0.683	-0.642	-0.779	-0.439	-0.523
	cc-pVTZ		-0.112	-0.214	-0.183	-0.324	0.042	-0.046
mDCNB	aug-cc-pVDZ	0.388	0.021	-0.061	-0.079	-0.252	0.100	0.030
	cc-pVDZ		-0.394	-0.513	-0.494	-0.706	-0.314	-0.420
	cc-pVTZ		0.054	-0.080	-0.061	-0.308	0.142	0.021
napthalenedione	aug-cc-pVDZ	1.257	-0.194	-0.214	-0.297	-0.435	-0.047	-0.064
	cc-pVDZ		-0.609	-0.678	-0.711	-0.888	-0.458	-0.526
	cc-pVTZ		-0.233	-0.282	-0.346	-0.421	-0.071	-0.107
NDCA	aug-cc-pVDZ	1.04	-0.160	-0.166	-0.268	-0.284	-0.053	-0.027
	cc-pVDZ		-0.520	-0.573	-0.628	-0.694	-0.410	-0.429
	cc-pVTZ		-0.127	-0.192	-0.251	-0.326	-0.008	-0.041
nitrobenzene	aug-cc-pVDZ	0.303	-0.105	-0.139	-0.203	-0.314	0.032	-0.007
	cc-pVDZ		-0.781	-0.865	-0.876	-1.015	-0.591	-0.654
	cc-pVTZ		-0.172	-0.290	-0.288	-0.507	-0.015	-0.137
nitrobenzonitrile	aug-cc-pVDZ	1.048	-0.011	-0.077	-0.123	-0.291	0.116	0.058
	cc-pVDZ		-0.559	-0.672	-0.668	-0.857	-0.398	-0.481
	cc-pVTZ		-0.005	-0.146	-0.134	-0.406	0.138	0.007
phenazine	aug-cc-pVDZ	0.897	-0.197	-0.188	-0.344	-0.543	-0.087	-0.058
	cc-pVDZ		-0.572	-0.602	-0.718	-0.916	-0.460	-0.468
	cc-pVTZ		-0.150	-0.207	-0.315	-0.684	-0.027	-0.064
phthalic anhydride	aug-cc-pVDZ	0.637	-0.046	-0.118	-0.144	-0.320	0.057	-0.008
	cc-pVDZ		-0.452	-0.583	-0.548	-0.784	-0.348	-0.472
	cc-pVTZ		-0.008	-0.147	-0.119	-0.391	0.104	-0.029
phthalimide	aug-cc-pVDZ	0.406	-0.141	-0.168	-0.233	-0.321	-0.034	-0.045
	cc-pVDZ		-0.600	-0.682	-0.690	-0.833	-0.490	-0.554
	cc-pVTZ		-0.129	-0.223	-0.235	-0.401	-0.011	-0.089
TCNE	aug-cc-pVDZ	2.758	0.124	-0.003	0.019	-0.181	0.249	0.140
	cc-pVDZ		-0.194	-0.364	-0.296	-0.538	-0.071	-0.222
	cc-pVTZ		0.244	0.070	0.126	-0.134	0.379	0.224
TCNQ	aug-cc-pVDZ	3.066	0.020	-0.059	-0.141	-0.392	0.149	0.105

Continued on next page

Molecule	Basis Set	CCSD(T)	$\Delta E_{EA} = E_{method} - E_{ref}$					
			fpCCD	fpCCSD	fpLCCD	fpLCCSD	CCD(pCCD)	CCSD(pCCD)
	cc-pVDZ		-0.258	-0.379	-0.420	-0.708	-0.131	-0.131
	cc-pVTZ		0.128	-0.001	-0.049	-0.378	0.269	0.175

Table S3: Energy differences in EAs for various pCCD-based DIP/IP-EOM flavors [eV] for the set of 22 organic acceptor molecules using various basis sets, that is $E_{\text{method}} = E_{\text{DIP}}^{\text{method}} - E_{\text{IP}}^{\text{method}}$. The experimental reference values are taken from Ref. 1.

Molecule	Basis Set	EXP	$\Delta E_{EA} = E_{method} - E_{ref}$					
			fpCCD	fpCCSD	fpLCCD	fpLCCSD	CCD(pCCD)	CCSD(pCCD)
acridine	aug-cc-pVDZ		-1.698	-1.664	-1.691	-1.633	-1.689	-1.645
	cc-pVDZ	0.9	-0.389	-0.452	-0.269	-0.600	-0.427	-0.487
	cc-pVTZ		0.087	-0.034	0.233	0.155	0.060	-0.077
anthracene	aug-cc-pVDZ		-1.357	-1.320	-1.349	-1.290	-1.348	-1.301
	cc-pVDZ	0.53	-0.237	-0.358	-0.110	-0.436	-0.256	-0.392
	cc-pVTZ		-0.033	-0.054	0.113	-0.008	-0.048	-0.063
azulene	aug-cc-pVDZ		-1.650	-1.603	-1.645	-1.556	-1.652	-1.569
	cc-pVDZ	0.8	-0.439	-0.474	-0.351	-0.371	-0.483	-0.521
	cc-pVTZ		-0.044	-0.099	0.065	0.028	-0.079	-0.141
benzonitrile	aug-cc-pVDZ		-0.822	-0.773	-0.815	-0.748	-0.825	-0.747
	cc-pVDZ	0.26	-0.778	-0.853	-0.705	-0.760	-0.823	-0.901
	cc-pVTZ		-0.279	-0.368	-0.186	-0.241	-0.322	-0.412
benzoquinone	aug-cc-pVDZ		-1.118	-0.908	-0.993	-1.250	-1.127	-0.823
	cc-pVDZ	1.85	-0.765	-0.797	-0.679	-4.394	-0.781	-0.812
	cc-pVTZ		-0.155	-0.196	-0.052	-0.528	-0.182	-0.232
dichlone	aug-cc-pVDZ		-0.117	-0.073	0.007	-0.112	-0.154	-0.103
	cc-pVDZ	2.21	-0.546	-0.611	-0.428	-0.578	-0.577	-0.634
	cc-pVTZ		-0.066	-0.128	0.079	-0.058	-0.091	-0.149
fumaronitrile	aug-cc-pVDZ		-0.152	-0.173	-0.096	0.053	-0.186	-0.187
	cc-pVDZ	1.25	-0.566	-0.687	-0.514	-0.632	-0.591	-0.700
	cc-pVTZ		0.020	-0.097	0.091	-0.017	-0.006	-0.114
maleic anhydride	aug-cc-pVDZ		-1.894	-1.873	-1.888	-1.859	-1.894	-1.858
	cc-pVDZ	1.44	-0.778	-0.895	-0.718	-0.835	-0.805	-1.440
	cc-pVTZ		-0.161	-0.289	-0.085	-0.209	-0.192	-0.340
mDCNB	aug-cc-pVDZ		-1.349	-1.290	-1.342	-1.250	-1.369	-1.235
	cc-pVDZ	0.91	-0.525	-0.623	-0.437	-0.531	-0.573	-0.639
	cc-pVTZ		-0.029	-0.133	0.079	-0.009	-0.071	-0.143
napthalenedione	aug-cc-pVDZ		-0.252	-0.163	-0.134	-0.089	-0.291	-0.192
	cc-pVDZ	1.81	-0.693	-0.717	-0.583	-0.766	-0.730	-0.750
	cc-pVTZ		-0.213	-0.237	-0.075	-0.218	-0.239	-0.257
nitrobenzene	aug-cc-pVDZ		-1.595	-1.544	-1.588	-1.516	-1.604	-1.512
	cc-pVDZ	1.00	-0.776	-0.909	-0.699	-0.925	-0.816	-0.980
	cc-pVTZ		-0.197	-0.341	-0.096	-0.412	-0.225	-0.407
nitrobenzonitrile	aug-cc-pVDZ		0.058	-0.024	0.148	0.217	0.013	-0.111
	cc-pVDZ	1.69	-0.518	-0.687	-0.434	-0.666	-0.564	-0.762
	cc-pVTZ		0.045	-0.132	0.153	1.035	0.009	-0.205
phenazine	aug-cc-pVDZ		0.116	0.085	0.242	0.483	0.070	0.018
	cc-pVDZ	1.31	-0.332	-0.416	-0.212	-0.419	-0.375	-0.470
	cc-pVTZ		0.121	0.014	0.267	0.290	0.092	-0.030
phthalic anhydride	aug-cc-pVDZ		-1.654	-1.617	-1.645	-1.602	-1.654	-1.593
	cc-pVDZ	1.25	-0.690	-0.780	-0.598	-0.568	-0.729	-0.820
	cc-pVTZ		-0.148	-0.253	-0.032	0.168	-0.180	-0.300
phthalimide	aug-cc-pVDZ		-1.662	-1.617	-1.657	-1.581	-1.688	-1.563
	cc-pVDZ	1.02	-0.758	-0.829	-0.666	-0.589	-0.799	-0.873
	cc-pVTZ		-0.200	-0.284	-0.084	0.336	-0.233	-0.334
TCNE	aug-cc-pVDZ		0.381	0.269	0.462	0.363	0.328	0.234
	cc-pVDZ	3.16	-0.111	-0.303	-0.042	-0.233	-0.156	-0.328
	cc-pVTZ		0.444	0.254	0.536	0.347	0.403	0.234
Cl_4benzoquinone	aug-cc-pVDZ		0.043	0.047	0.145	-0.067	0.010	0.001
	cc-pVDZ	2.78	-0.517	-0.617	-0.421	-0.957	-0.532	-0.632
	cc-pVTZ		-0.012	-0.104	0.114	-0.112	-0.021	-0.117
Cl_4isobenzofuranedione	aug-cc-pVDZ		0.074	0.029	0.182	0.247	0.029	-0.024

Continued on next page

Molecule	Basis Set	EXP	$\Delta E_{EA} = E_{method} - E_{ref}$					
			fpCCD	fpCCSD	fpLCCD	fpLCCSD	CCD(pCCD)	CCSD(pCCD)
	cc-pVDZ	1.96	-0.360	-0.504	-0.264	-0.144	-0.395	-0.544
	cc-pVTZ		0.078	-0.047	0.204	-0.636	0.058	-0.074
F_4benzenedicarbonitrile	aug-cc-pVDZ		-2.648	-2.471	-2.626	-1.460	-2.641	-2.393
	cc-pVDZ	1.89	-0.324	-0.528	-0.247	-0.462	-0.375	-0.573
	cc-pVTZ		0.190	0.000	0.290	0.086	0.146	-0.042
F_4benzoquinone	aug-cc-pVDZ		-0.153	-0.145	-0.054	-6.365	-0.186	-0.194
	cc-pVDZ	2.7	-0.809	-0.935	-0.720	-2.387	-0.832	-0.966
	cc-pVTZ		-0.225	-0.327	-0.114	-2.346	-0.251	-0.368
dinitrobenzonitrile	aug-cc-pVDZ		-0.814	-0.594	-0.722	-5.566	-0.889	-0.301
	cc-pVDZ	2.16	-1.374	-1.251	-1.288	4.035	-1.522	-0.982
	cc-pVTZ		-0.787	-0.695	-0.678	-3.380	-0.905	-0.423
TCNQ	aug-cc-pVDZ		0.889	0.806	0.987	0.910	0.848	0.778
	cc-pVDZ	2.8	0.487	0.343	0.573	0.471	0.453	0.324
	cc-pVTZ		0.949	0.798	1.061	0.502	0.925	0.788

Table S4: Energy differences in EAs for various pCCD-based DIP/IP-EOM flavors [eV] for the set of 24 organic acceptor molecules using various basis sets, that is $E_{\text{method}} = E_{\text{DIP}}^{\text{method}} - E_{\text{IP}}^{\text{method}}$. The Δ-CCSD(T) reference values are taken from Refs. 1,3 and determined for the aug-cc-pVDZ basis set only.

Molecule	Basis Set	CCSD(T)	$\Delta E_{EA} = E_{method} - E_{ref}$					
			fpCCD	fpCCSD	fpLCCD	fpLCCSD	CCD(pCCD)	CCSD(pCCD)
acridine	aug-cc-pVDZ	0.48	-1.278	-1.244	-1.271	-1.213	-1.269	-1.225
	cc-pVDZ		0.031	-0.032	0.151	-0.180	-0.007	-0.067
	cc-pVTZ		0.507	0.386	0.653	0.575	0.480	0.343
anthracene	aug-cc-pVDZ	0.141	-0.968	-0.931	-0.960	-0.901	-0.959	-0.912
	cc-pVDZ		0.152	0.031	0.279	-0.047	0.133	-0.003
	cc-pVTZ		0.356	0.335	0.502	0.381	0.341	0.326
azulene	aug-cc-pVDZ	0.369	-1.219	-1.172	-1.214	-1.125	-1.221	-1.138
	cc-pVDZ		-0.008	-0.043	0.080	0.060	-0.052	-0.090
	cc-pVTZ		0.387	0.332	0.496	0.459	0.352	0.290
benzonitrile	aug-cc-pVDZ	-0.431	-0.131	-0.082	-0.124	-0.057	-0.134	-0.056
	cc-pVDZ		-0.087	-0.162	-0.014	-0.069	-0.132	-0.210
	cc-pVTZ		0.412	0.323	0.505	0.450	0.369	0.279
benzoquinone	aug-cc-pVDZ	1.323	-0.591	-0.381	-0.466	-0.723	-0.600	-0.296
	cc-pVDZ		-0.238	-0.270	-0.152	-3.867	-0.254	-0.285
	cc-pVTZ		0.372	0.331	0.475	-0.001	0.345	0.295
dichlone	aug-cc-pVDZ	1.686	0.407	0.451	0.531	0.412	0.370	0.421
	cc-pVDZ		-0.022	-0.087	0.096	-0.054	-0.053	-0.110
	cc-pVTZ		0.458	0.396	0.603	0.466	0.433	0.375
fumaronitrile	aug-cc-pVDZ	0.728	0.370	0.349	0.426	0.575	0.336	0.335
	cc-pVDZ		-0.044	-0.165	0.008	-0.110	-0.069	-0.178
	cc-pVTZ		0.542	0.425	0.613	0.505	0.516	0.408
maleic anhydride	aug-cc-pVDZ	0.773	-1.227	-1.206	-1.221	-1.192	-1.227	-1.191
	cc-pVDZ		-0.111	-0.228	-0.051	-0.168	-0.138	-0.773
	cc-pVTZ		0.506	0.378	0.582	0.458	0.475	0.327
mDCNB	aug-cc-pVDZ	0.388	-0.827	-0.768	-0.820	-0.728	-0.847	-0.713
	cc-pVDZ		-0.003	-0.101	0.085	-0.009	-0.051	-0.117
	cc-pVTZ		0.493	0.389	0.601	0.513	0.451	0.379
napthalenedione	aug-cc-pVDZ	1.257	0.301	0.390	0.419	0.464	0.262	0.361
	cc-pVDZ		-0.140	-0.164	-0.030	-0.213	-0.177	-0.197
	cc-pVTZ		0.340	0.316	0.478	0.335	0.314	0.296
nitrobenzene	aug-cc-pVDZ	0.303	-0.898	-0.847	-0.891	-0.819	-0.907	-0.815
	cc-pVDZ		-0.079	-0.212	-0.002	-0.228	-0.119	-0.283
	cc-pVTZ		0.500	0.356	0.601	0.285	0.472	0.290
nitrobenzonitrile	aug-cc-pVDZ	1.048	0.700	0.618	0.790	0.859	0.655	0.531
	cc-pVDZ		0.124	-0.045	0.208	-0.024	0.078	-0.120
	cc-pVTZ		0.687	0.510	0.795	1.677	0.651	0.437
phenazine	aug-cc-pVDZ	0.897	0.529	0.498	0.655	0.896	0.483	0.431
	cc-pVDZ		0.081	-0.003	0.201	-0.006	0.038	-0.057

Continued on next page

Molecule	Basis Set	CCSD(T)	$\Delta E_{\mathrm{EA}} = E_{\mathrm{method}} - E_{\mathrm{ref}}$					
			fpCCD	fpCCSD	fpLCCD	fpLCCSD	CCD(pCCD)	CCSD(pCCD)
	cc-pVTZ		0.534	0.427	0.680	0.703	0.505	0.383
phthalic anhydrid	aug-cc-pVDZ	0.637	-1.041	-1.004	-1.032	-0.989	-1.041	-0.980
	cc-pVDZ		-0.077	-0.167	0.015	0.045	-0.116	-0.207
	cc-pVTZ		0.465	0.360	0.581	0.781	0.433	0.313
phthalimide	aug-cc-pVDZ	0.406	-1.048	-1.003	-1.043	-0.967	-1.074	-0.949
	cc-pVDZ		-0.144	-0.215	-0.052	0.025	-0.185	-0.259
	cc-pVTZ		0.414	0.330	0.530	0.950	0.381	0.280
TCNE	aug-cc-pVDZ	2.758	0.783	0.671	0.864	0.765	0.730	0.636
	cc-pVDZ		0.291	0.099	0.360	0.169	0.246	0.074
	cc-pVTZ		0.846	0.656	0.938	0.749	0.805	0.636
Cl_4benzoquinone	aug-cc-pVDZ	2.224	0.599	0.603	0.701	0.489	0.566	0.557
	cc-pVDZ		0.039	-0.061	0.135	-0.401	0.024	-0.076
	cc-pVTZ		0.544	0.452	0.670	0.444	0.535	0.439
Cl_4isobenzofuranedione	aug-cc-pVDZ	1.411	0.623	0.578	0.731	0.796	0.578	0.525
	cc-pVDZ		0.189	0.045	0.285	0.405	0.154	0.005
	cc-pVTZ		0.627	0.502	0.753	-0.087	0.607	0.475
F_4benzenedicarbonitrile	aug-cc-pVDZ	1.334	-2.092	-1.915	-2.070	-0.904	-2.085	-1.837
	cc-pVDZ		0.232	0.028	0.309	0.094	0.181	-0.017
	cc-pVTZ		0.746	0.556	0.846	0.642	0.702	0.514
F_4benzoquinone	aug-cc-pVDZ	2.015	0.532	0.540	0.631	-5.680	0.499	0.491
	cc-pVDZ		-0.124	-0.250	-0.035	-1.702	-0.147	-0.281
	cc-pVTZ		0.460	0.358	0.571	-1.661	0.434	0.317
NDCA	aug-cc-pVDZ	1.04	-1.524	-1.458	-1.518	-1.398	-1.532	-1.409
	cc-pVDZ		0.050	-0.069	0.161	0.219	0.009	-0.110
	cc-pVTZ		0.467	0.346	0.603	0.146	0.440	0.313
bodipy	aug-cc-pVDZ	1.472	0.289	0.100	0.380	0.386	0.232	0.008
	cc-pVDZ		0.144	0.061	0.233	0.151	0.123	0.029
	cc-pVTZ		0.508	0.414	0.616	0.521	0.490	0.384
dinitrobenzonitrile	aug-cc-pVDZ	1.521	-0.175	0.045	-0.083	-4.927	-0.250	0.338
	cc-pVDZ		-0.735	-0.612	-0.649	4.674	-0.883	-0.343
	cc-pVTZ		-0.148	-0.056	-0.039	-2.741	-0.266	0.216
TCNQ	aug-cc-pVDZ	3.066	0.623	0.540	0.721	0.644	0.582	0.512
	cc-pVDZ		0.221	0.077	0.307	0.205	0.187	0.058
	cc-pVTZ		0.683	0.532	0.795	0.236	0.659	0.522

Table S5: Energy differences in EAs for various pCCD-based EA-EOM flavors [eV] for the set of 22 organic acceptor molecules extrapolated to the basis set limit (CBS). The experimental reference values are taken from Ref. 1.

Molecule	EXP	$\Delta E_{\mathrm{EA}} = E^{\mathrm{CBS}}_{\mathrm{method}} - E_{\mathrm{ref}}$					
		fpCCD	fpCCSD	fpLCCD	fpLCCSD	CCD(pCCD)	CCSD(pCCD)
acridine	0.9	-0.401	-0.459	-0.557	-0.712	-0.284	-0.322
anthracene	0.53	-0.385	-0.447	-0.535	-0.849	-0.267	-0.305
azulene	0.8	-0.314	-0.439	-0.458	-0.374	-0.204	-0.323
benzonitrile	0.26	-0.526	-0.654	-0.639	-0.869	-0.435	-0.593
benzoquinone	1.85	-0.543	-0.606	-0.649	-0.759	-0.361	-0.414
dichlone	2.21	-0.435	-1.172	-1.239	-1.282	-0.953	-0.972
dinitrobenzonitrile	2.16	-0.370	-0.559	-0.499	-0.870	-0.233	-0.418
fumaronitrile	1.25	-0.338	-0.449	-0.421	-0.566	-0.192	-0.290
Maleic_Anhydride	1.44	-0.581	-0.684	-0.657	-0.799	-0.422	-0.511
mDCNB	0.91	-0.280	-0.420	-0.401	-0.663	-0.189	-0.315
napthalenedione	1.81	-0.628	-0.668	-0.745	-0.776	-0.462	-0.483
nitrobenzene	1.0	-0.612	-0.744	-0.737	-0.990	-0.469	-0.616
nitrobenzonitrile	1.69	-0.414	-0.567	-0.552	-0.858	-0.278	-0.430
phenazine	1.31	-0.385	-0.453	-0.558	-1.000	-0.257	-0.306
phthalic_anhydrid	1.25	-0.434	-0.577	-0.552	-0.838	-0.319	-0.455
phthalimide	1.02	-0.545	-0.644	-0.657	-0.833	-0.423	-0.507
TCNE	3.16	0.027	-0.150	-0.099	-0.366	0.167	0.010
TCNQ	2.8	0.557	0.424	0.374	0.027	0.704	0.570
Cl_4benzoquinone	2.78	-0.462	-0.589	-0.580	-0.785	-0.277	-0.393
Cl_4isobenzofuranedione	1.96	-0.324	-0.491	-0.445	-0.732	-0.189	-0.334

Continued on next page

Molecule	EXP	$\Delta E_{EA} = E^{CBS}_{method} - E_{ref}$					
		fpCCD	fpCCSD	fpLCCD	fpLCCSD	CCD(pCCD)	CCSD(pCCD)
F_4benzenedicarbonitrile	1.89	-0.122	-0.318	-0.265	-0.687	-0.027	-0.210
F_4benzoquinone	2.7	-0.574	-0.698	-0.691	-0.892	-0.389	-0.498

Table S6: Energy differences in EAs for various pCCD-based DIP/IP-EOM flavors [eV] for the set of 22 organic acceptor molecules extrapolated to the basis set limit (CBS), that is $E_{\text{method}} = E^{\text{method}}_{\text{DIP}} - E^{\text{method}}_{\text{IP}}$. The experimental reference values are taken from Ref. 1.

Molecule	EXP	$\Delta E_{EA} = E^{CBS}_{method} - E_{ref}$					
		fpCCD	fpCCSD	fpLCCD	fpLCCSD	CCD(pCCD)	CCSD(pCCD)
acridine	0.9	0.288	0.142	0.445	0.473	0.265	0.095
anthracene	0.53	0.053	0.074	0.206	0.172	0.040	0.076
azulene	0.8	0.123	0.059	0.240	0.196	0.091	0.020
benzonitrile	0.26	-0.068	-0.163	0.032	-0.023	-0.111	-0.206
benzoquinone	1.85	0.103	0.058	0.212	1.099	0.071	0.012
dichlone	2.21	0.136	0.075	0.292	0.161	0.114	0.055
dinitrobenzonitrile	2.16	-0.540	-0.461	-0.421	-6.503	-0.646	-0.188
fumaronitrile	1.25	0.267	0.152	0.346	0.241	0.241	0.133
Maleic_Anhydride	1.44	0.099	-0.033	0.182	0.054	0.066	0.124
mDCNB	0.91	0.179	0.074	0.296	0.210	0.141	0.066
napthalenedione	1.81	-0.012	-0.034	0.139	0.013	-0.033	-0.049
nitrobenzene	1.0	0.048	-0.103	0.158	-0.196	0.025	-0.166
nitrobenzonitrile	1.69	0.282	0.101	0.400	1.751	0.251	0.029
phenazine	1.31	0.312	0.195	0.469	0.588	0.289	0.155
phthalic_anhydrid	1.25	0.081	-0.030	0.207	0.478	0.051	-0.081
phthalimide	1.02	0.036	-0.055	0.161	0.726	0.005	-0.107
TCNE	3.16	0.677	0.489	0.780	0.592	0.639	0.470
TCNQ	2.8	1.144	0.990	1.267	0.515	1.124	0.983
Cl_4benzoquinone	2.78	0.201	0.112	0.340	0.244	0.194	0.100
Cl_4isobenzofuranedione	1.96	0.263	0.145	0.402	-0.843	0.250	0.124
F_4benzenedicarbonitrile	1.89	0.406	0.222	0.516	0.317	0.365	0.182
F_4benzoquinone	2.7	0.021	-0.071	0.141	-2.329	-0.006	-0.117

Table S7: Energy differences in EAs for various pCCD-based EA-EOM flavors [eV] for the set of 24 organic acceptor molecules extrapolated to the basis set limit (CBS). The Δ-CCSD(T) reference values are taken from Ref. 1,3 and determined for the aug-cc-pVDZ basis set only.

Molecule	CCSD(T)	$\Delta E_{EA} = E^{CBS}_{method} - E_{ref}$					
		fpCCD	fpCCSD	fpLCCD	fpLCCSD	CCD(pCCD)	CCSD(pCCD)
acridine	0.48	0.019	-0.039	-0.137	-0.292	0.136	0.098
anthracene	0.141	0.004	-0.058	-0.146	-0.460	0.122	0.084
azulene	0.369	0.117	-0.008	-0.027	0.057	0.227	0.108
benzonitrile	-0.431	0.165	0.037	0.052	-0.178	0.256	0.098
benzoquinone	1.323	-0.016	-0.079	-0.122	-0.232	0.166	0.113
bodipy	1.472	0.092	0.059	-0.061	-0.043	0.133	0.111
dichlone	1.686	0.089	-0.648	-0.715	-0.758	-0.429	-0.448
dinitrobenzonitrile	1.521	0.269	0.080	0.140	-0.231	0.406	0.221
fumaronitrile	0.728	0.184	0.073	0.101	-0.044	0.330	0.232
Maleic_Anhydride	0.773	0.086	-0.017	0.010	-0.132	0.245	0.156
mDCNB	0.388	0.242	0.102	0.121	-0.141	0.333	0.207
napthalenedione	1.257	-0.075	-0.115	-0.192	-0.223	0.091	0.070
NDCA	1.04	0.038	-0.032	-0.092	-0.171	0.161	0.123
nitrobenzene	0.303	0.085	-0.047	-0.040	-0.293	0.228	0.081
nitrobenzonitrile	1.048	0.228	0.075	0.090	-0.216	0.364	0.212
phenazine	0.897	0.028	-0.040	-0.145	-0.587	0.156	0.107
phthalic_anhydrid	0.637	0.179	0.036	0.061	-0.225	0.294	0.158
phthalimide	0.406	0.069	-0.030	-0.043	-0.219	0.191	0.107

Continued on next page

Molecule	CCSD(T)	$\Delta E_{EA} = E^{CBS}_{method} - E_{ref}$					
		fpCCD	fpCCSD	fpLCCD	fpLCCSD	CCD(pCCD)	CCSD(pCCD)
TCNE	2.758	0.429	0.252	0.303	0.036	0.569	0.412
TCNQ	3.066	0.291	0.158	0.108	-0.239	0.438	0.304
Cl_4benzoquinone	2.224	0.094	-0.033	-0.024	-0.229	0.279	0.163
Cl_4isobenzofuranedione	1.411	0.225	0.058	0.104	-0.183	0.360	0.215
F_4benzenedicarbonitrile	1.334	0.434	0.238	0.291	-0.131	0.529	0.346
F_4benzoquinone	2.015	0.111	-0.013	-0.006	-0.207	0.296	0.187

Table S8: Energy differences in EAs for various pCCD-based DIP/IP-EOM flavors [eV] for the set of 24 organic acceptor molecules extrapolated to the basis set limit (CBS), that is $E_{\text{method}} = E^{\text{method}}_{\text{DIP}} - E^{\text{method}}_{\text{IP}}$. The Δ-CCSD(T) reference values are taken from Ref. 1,3 and determined for the aug-cc-pVDZ basis set only.

Molecule	CCSD(T)	$\Delta E_{EA} = E^{CBS}_{method} - E_{ref}$					
		fpCCD	fpCCSD	fpLCCD	fpLCCSD	CCD(pCCD)	CCSD(pCCD)
acridine	0.48	0.708	0.562	0.865	0.893	0.685	0.515
anthracene	0.141	0.442	0.463	0.595	0.561	0.429	0.465
azulene	0.369	0.554	0.490	0.671	0.627	0.522	0.451
benzonitrile	-0.431	0.623	0.528	0.723	0.668	0.580	0.485
benzoquinone	1.323	0.630	0.585	0.739	1.626	0.598	0.539
bodipy	1.472	0.662	0.563	0.777	0.676	0.645	0.533
dichlone	1.686	0.660	0.599	0.816	0.685	0.638	0.579
dinitrobenzonitrile	1.521	0.099	0.178	0.218	-5.864	-0.007	0.451
fumaronitrile	0.728	0.789	0.674	0.868	0.763	0.763	0.655
Maleic_Anhydride	0.773	0.766	0.634	0.849	0.721	0.733	0.791
mDCNB	0.388	0.701	0.596	0.818	0.732	0.663	0.588
napthalenedione	1.257	0.541	0.519	0.692	0.566	0.520	0.504
NDCA	1.04	0.643	0.521	0.789	0.116	0.622	0.491
nitrobenzene	0.303	0.745	0.594	0.855	0.501	0.722	0.531
nitrobenzonitrile	1.048	0.924	0.743	1.042	2.393	0.893	0.671
phenazine	0.897	0.725	0.608	0.882	1.001	0.702	0.568
phthalic_anhydrid	0.637	0.694	0.583	0.820	1.091	0.664	0.532
phthalimide	0.406	0.650	0.559	0.775	1.340	0.619	0.507
TCNE	2.758	1.079	0.891	1.182	0.994	1.041	0.872
TCNQ	3.066	0.878	0.724	1.001	0.249	0.858	0.717
Cl_4benzoquinone	2.224	0.757	0.668	0.896	0.800	0.750	0.656
Cl_4isobenzofuranedione	1.411	0.812	0.694	0.951	-0.294	0.799	0.673
F_4benzenedicarbonitrile	1.334	0.962	0.778	1.072	0.873	0.921	0.738
F_4benzoquinone	2.015	0.706	0.614	0.826	-1.644	0.679	0.568

Table S9: Statistical error metrics (ME, MAE, RMSE, MPE, SD) [eV] for computed electron affinities (EAs) of 24 organic acceptor molecules using various methods and basis sets, relative to Δ-CCSD(T) reference values determined with the aug-cc-pVDZ basis set from Refs. 1,3. Values marked with * are extrapolated to the complete basis set limit, as described in Ref. 4. CCD(pCCD) and CCSD(pCCD) refer to CCD and CCSD methods performed using pCCD-optimized natural orbitals or the pCCD reference determinant.

Method	Basis Set	Errors w.r.t. Δ-CCSD(T) ($E_{method} - E_{ref}$)				
		ME [eV]	MAE [eV]	RMSE [eV]	MPE [%]	SD [eV]
EA-EOM-fpCCD	aug-cc-pVDZ	−0.108	0.139	0.239	23.8	0.218
	cc-pVDZ	−0.480	0.480	0.502	69.2	0.150
	cc-pVTZ	−0.043	0.109	0.133	16.1	0.128
	CBS*	0.141	0.149	0.190	13.4	0.129
EA-EOM-fpCCSD	aug-cc-pVDZ	−0.155	0.158	0.247	26.9	0.196

Continued on next page

Method	Basis Set	Errors w.r.t. Δ-CCSD(T) ($E_{method} - E_{ref}$)				
		ME [eV]	MAE [eV]	RMSE [eV]	MPE [%]	SD [eV]
	cc-pVDZ	−0.579	0.579	0.592	79.4	0.126
	cc-pVTZ	−0.171	0.182	0.218	25.7	0.137
	CBS*	0.000	0.097	0.163	9.4	0.166
EA-EOM-fpLCCD	aug-cc-pVDZ	−0.206	0.212	0.287	36.5	0.204
	cc-pVDZ	−0.586	0.586	0.603	83.8	0.143
	cc-pVTZ	−0.184	0.205	0.245	30.5	0.165
	CBS*	−0.015	0.131	0.194	15.3	0.197
EA-EOM-fpLCCSD	aug-cc-pVDZ	−0.313	0.319	0.369	49.2	0.201
	cc-pVDZ	−0.730	0.730	0.748	101.2	0.169
	cc-pVTZ	−0.376	0.376	0.407	53.5	0.159
	CBS*	−0.223	0.230	0.282	35.8	0.178
EA-EOM-CCD(pCCD)	aug-cc-pVDZ	0.005	0.127	0.232	18.1	0.237
	cc-pVDZ	−0.357	0.357	0.380	53.0	0.131
	cc-pVTZ	0.067	0.121	0.168	10.6	0.158
	CBS*	0.245	0.281	0.308	30.2	0.191
EA-EOM-CCSD(pCCD)	aug-cc-pVDZ	−0.032	0.085	0.214	13.7	0.216
	cc-pVDZ	−0.432	0.432	0.445	61.5	0.111
	cc-pVTZ	−0.026	0.083	0.126	9.7	0.126
	CBS*	0.144	0.182	0.208	19.1	0.154
DIP/IP-EOM-fpCCD	aug-cc-pVDZ	−0.303	0.782	0.904	128.8	0.870
	cc-pVDZ	−0.011	0.140	0.202	15.1	0.206
	cc-pVTZ	0.488	0.500	0.520	61.3	0.184
	CBS*	0.698	0.698	0.722	85.3	0.189
DIP/IP-EOM-fpCCSD	aug-cc-pVDZ	−0.276	0.725	0.850	122.3	0.822
	cc-pVDZ	−0.106	0.134	0.184	14.6	0.154
	cc-pVTZ	0.390	0.395	0.410	49.5	0.129
	CBS*	0.599	0.599	0.613	74.7	0.133
DIP/IP-EOM-fpLCCD	aug-cc-pVDZ	−0.244	0.815	0.924	130.7	0.910
	cc-pVDZ	0.080	0.162	0.220	21.0	0.209
	cc-pVTZ	0.602	0.605	0.628	76.8	0.182
	CBS*	0.822	0.822	0.841	102.6	0.184
DIP/IP-EOM-fpLCCSD	aug-cc-pVDZ	−0.639	1.163	1.734	147.0	1.647
	cc-pVDZ	−0.043	0.547	1.297	41.8	1.324
	cc-pVTZ	0.283	0.657	0.881	76.5	0.853
	CBS*	0.420	1.070	1.542	120.5	1.516
DIP/IP-EOM-CCD(pCCD)	aug-cc-pVDZ	−0.327	0.768	0.896	128.0	0.852
	cc-pVDZ	−0.050	0.148	0.224	16.1	0.223
	cc-pVTZ	0.455	0.477	0.494	58.3	0.197
	CBS*	0.668	0.669	0.696	82.1	0.198
DIP/IP-EOM-CCSD(pCCD)	aug-cc-pVDZ	−0.266	0.694	0.817	118.1	0.789
	cc-pVDZ	−0.151	0.165	0.229	19.0	0.177
	cc-pVTZ	0.368	0.368	0.380	45.7	0.097
	CBS*	0.587	0.587	0.597	72.7	0.112
EA-EOM-CCSD	aug-cc-pVDZ	0.019	0.046	0.062	4.7	0.060
CC2	aug-cc-pVDZ	0.472	0.472	0.485	58.1	0.112
ADC(2)	aug-cc-pVDZ	0.524	0.524	0.542	62.7	0.142
SCS-ADC(2)	aug-cc-pVDZ	0.152	0.157	0.196	16.1	0.127
SOS-ADC(2)	aug-cc-pVDZ	−0.036	0.112	0.130	13.7	0.128
Exp.	–	0.516	0.540	0.551	68.9	0.200

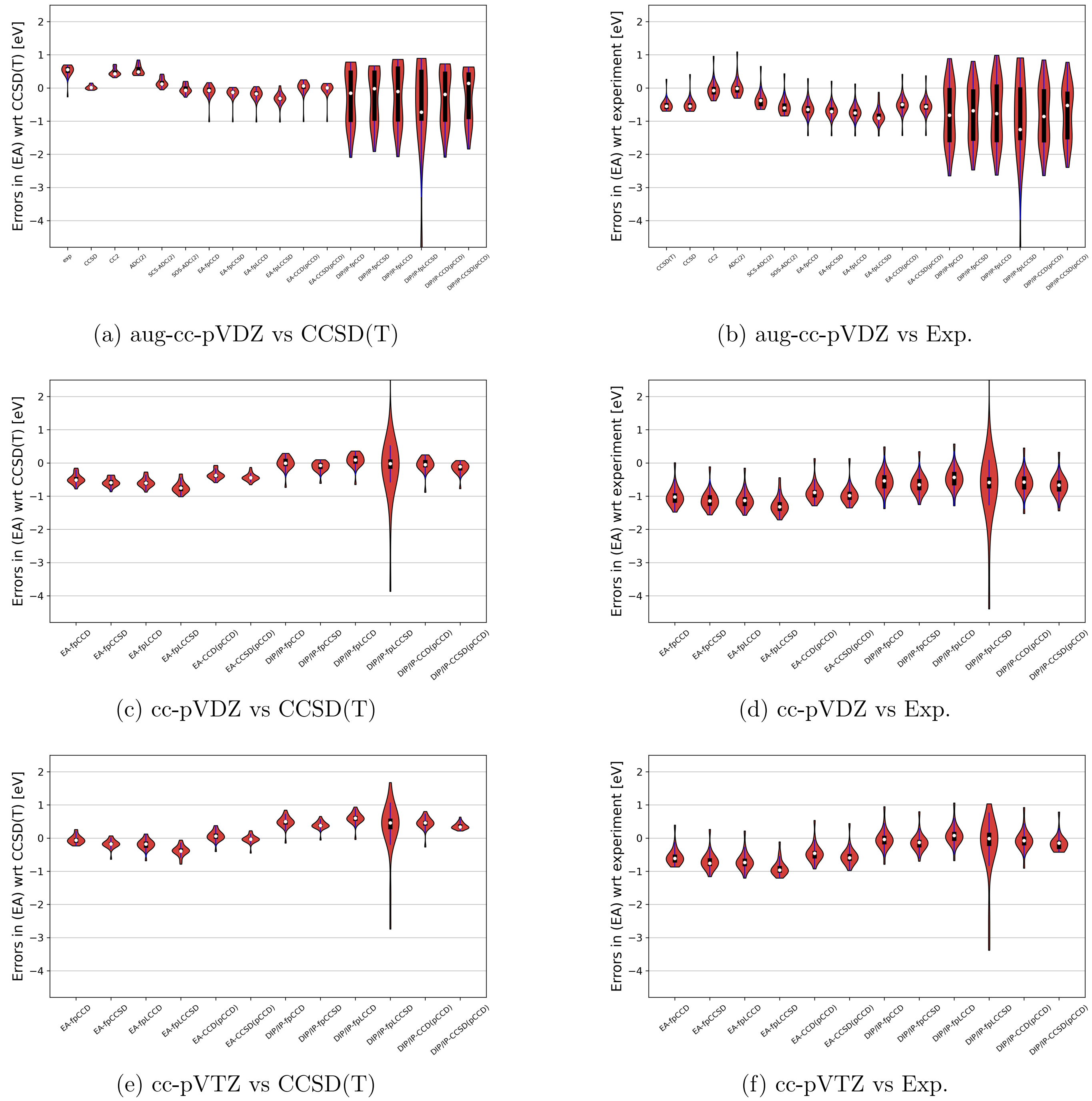

(a) aug-cc-pVDZ vs CCSD(T)

(b) aug-cc-pVDZ vs Exp.

(c) cc-pVDZ vs CCSD(T)

(d) cc-pVDZ vs Exp.

(e) cc-pVTZ vs CCSD(T)

(f) cc-pVTZ vs Exp.

Figure S1: Violin plots showing EA errors [eV] for small organic molecules across three basis sets. The left column shows errors relative to Δ-CCSD(T)/aug-cc-pVDZ; the right column shows errors relative to experiment. White dots denote medians. For clarity, the EOM prefix is omitted from method names. See Tables S1–S4 for numerical values.

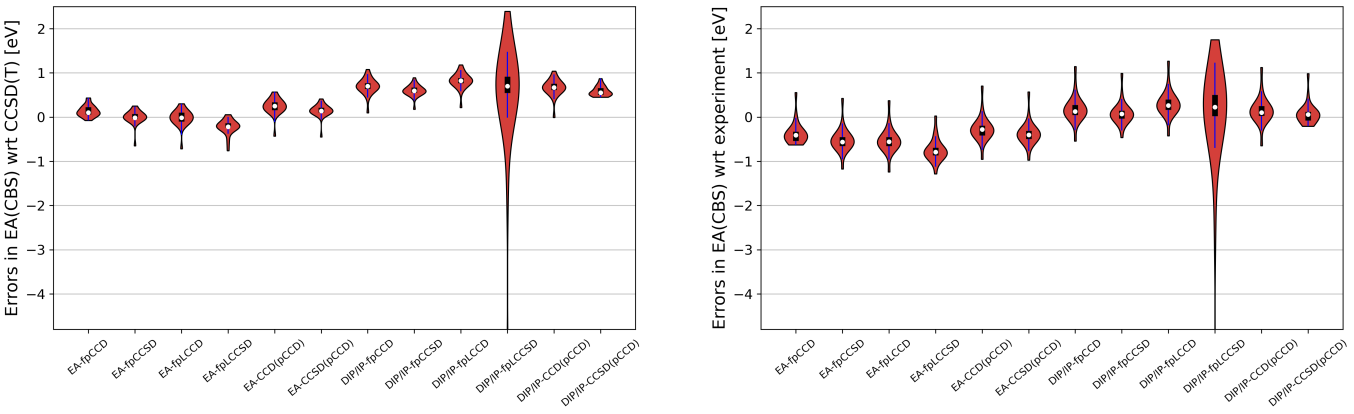

(a) CBS extrapolated EAs vs Δ-CCSD(T) (b) CBS extrapolated EAs vs Experiment

Figure S2: Violin plots showing electron affinity errors [eV] computed using CBS extrapolated energies. Left panel compares errors relative to Δ-CCSD(T)/aug-cc-pVDZ, while the right panel compares errors relative to experimental reference values. White dots denote medians. The violin plots visualize the full error distribution using kernel density estimation. See Tables S5–S8 for numerical values.

S3 Effect of the Cholesky decomposition (CD) threshold

We evaluated the impact of the Cholesky decomposition threshold (CD) on (i) total coupled-cluster energies of the neutral species and (ii) electron affinities (EAs) obtained either directly from EA-EOM-CC or indirectly via DIP/IP-EOM-CC as $E_{\mathrm{EA}}^{\mathrm{DIP/IP}} = E_{\mathrm{DIP}} - E_{\mathrm{IP}}$. For each molecule and basis set, we report results for CD thresholds of 10^{-4} (default) and 10^{-5}. Total energies are in E_h, while EAs are in units of eV.

Table S10: Sensitivity of energies to the CD threshold for selected molecule and basis sets for orbital-optimized pCCD and various fpCCSD frameworks. E_{pCCD} [E_h] and E_{CC} [E_h] are total energies (neutral); $E_{\mathrm{EA}}^{\mathrm{CC}}$ is the EA from EA-EOM-fpCCSD [eV]; E_{IP} [E_h] and E_{DIP} [E_h] are the total energies of IP- and DIP-EOM-fpCCSD; and $E_{\mathrm{EA}}^{\mathrm{DIP/IP}} = E_{\mathrm{DIP}} - E_{\mathrm{IP}}$ [eV]. nc: not computed.

**Molecule**	**Basis Set**	**CD**	E_{pCCD}	E_{CC}	$E_{\mathrm{EA}}^{\mathrm{CC}}$	E_{IP}	E_{DIP}	$E_{\mathrm{EA}}^{\mathrm{DIP/IP}}$
azulene	aug-cc-pVDZ	10^{-4}	-383.803336	-384.743237	0.2760	-384.712488	-384.742034	-0.8040
		10^{-5}	-383.803579	-384.743371	0.2756	-384.712720	-384.742240	-0.8033
	cc-pVTZ	10^{-4}	-383.964772	-385.028250	0.2119	-385.033530	-385.007768	0.7010
		10^{-5}	-383.964729	-385.028210	0.2120	-385.033707	-385.007936	0.7013
benzonitrile	aug-cc-pVDZ	10^{-4}	-322.858722	-323.613278	-0.4240	-323.591926	-323.610778	-0.5130
		10^{-5}	-322.858727	-323.613285	-0.4236	-323.592173	-323.610978	-0.5117
	cc-pVTZ	10^{-4}	-322.988862	-323.849520	-0.5785	-323.823152	-323.827121	-0.1080
		10^{-5}	-322.988891	-323.849549	-0.5785	-323.823381	-323.827331	-0.1075
benzoquinone	aug-cc-pVDZ	10^{-4}	-379.719941	-380.507851	1.0950	nc	nc	nc
		10^{-5}	-379.719937	-380.507852	1.0953	nc	nc	nc
	cc-pVTZ	10^{-4}	-379.863885	-380.781080	1.0494	-380.814158	-380.753338	1.6550
		10^{-5}	-379.863932	-380.781121	1.0495	-380.814406	-380.753603	1.6545

Continued on next page

Molecule	Basis Set	CD	E_{pCCD}	E_{CC}	$E_{\text{EA}}^{\text{CC}}$	E_{IP}	E_{DIP}	$E_{\text{EA}}^{\text{DIP/IP}}$
fumaronitrile	aug-cc-pVDZ	10^{-4}	-261.838886	-262.405031	0.6190	-262.422083	-262.382504	1.0770
		10^{-5}	-261.838892	-262.405047	0.6190	-262.422252	-262.382648	1.0777
	cc-pVTZ	10^{-4}	-261.936337	-262.589895	0.5993	-262.604433	-262.562024	1.1540
		10^{-5}	-261.936343	-262.589900	0.5994	-262.604576	-262.562188	1.1534
Maleic Anhydride	aug-cc-pVDZ	10^{-4}	-377.658451	-378.408893	0.6050	-378.390174	-378.406086	-0.4330
		10^{-5}	-377.658449	-378.408891	0.6054	-378.390379	-378.406288	-0.4329
	cc-pVTZ	10^{-4}	-377.794342	-378.679930	0.5588	-378.692474	-378.650175	1.1510
		10^{-5}	-377.794403	-378.679982	0.5588	-378.692784	-378.650453	1.1519
mDCNB	aug-cc-pVDZ	10^{-4}	-414.682020	-415.634163	0.3270	-415.617220	-415.631185	-0.3800
		10^{-5}	-414.682046	-415.634192	0.3268	-415.617800	-415.631343	-0.3685
	cc-pVTZ	10^{-4}	-414.840723	-415.928892	0.3076	-415.932382	-415.903791	0.7780
		10^{-5}	-414.840804	-415.928975	0.3075	-415.932678	-415.904116	0.7772
napthalenedione	aug-cc-pVDZ	10^{-4}	-532.560230	-533.738905	1.0430	nc	nc	nc
		10^{-5}	-532.560234	-533.738915	1.0434	nc	nc	nc
	cc-pVTZ	10^{-4}	-532.763890	-534.120550	0.9752	nc	nc	nc
		10^{-5}	-532.763923	-534.120577	0.9753	nc	nc	nc
nitrobenzene	aug-cc-pVDZ	10^{-4}	-434.693007	-435.660584	0.1640	-435.637028	-435.657020	-0.5440
		10^{-5}	-434.693029	-435.660608	0.1643	-435.637309	-435.657267	-0.5431
	cc-pVTZ	10^{-4}	-434.856909	-435.970549	0.0134	-435.966155	-435.941937	0.6590
		10^{-5}	-434.856941	-435.970571	0.0135	-435.966404	-435.942213	0.6583
phthalic anhydrid	aug-cc-pVDZ	10^{-4}	-530.498281	-531.636680	0.5190	-531.619341	-531.632828	-0.3670
		10^{-5}	-530.498308	-531.636713	no data	-531.619691	-531.633143	-0.3660
	cc-pVTZ	10^{-4}	-530.695800	-532.017712	0.4898	nc	nc	nc
		10^{-5}	-530.695850	-532.017757	0.4898	nc	nc	nc
phthalimide	aug-cc-pVDZ	10^{-4}	-510.676636	-511.802091	0.2380	-511.778354	-511.800294	-0.5970
		10^{-5}	-510.676679	-511.802137	0.2384	-511.778718	-511.800628	-0.5962
	cc-pVTZ	10^{-4}	-510.868890	-512.168441	0.1826	nc	nc	nc
		10^{-5}	-510.868930	-512.168476	0.1827	nc	nc	nc
TCNE	aug-cc-pVDZ	10^{-4}	-445.450976	-446.417757	2.7550	-446.509898	-446.383885	3.4290
		10^{-5}	-445.451009	-446.417806	2.7546	-446.510213	-446.384194	3.4292
	cc-pVTZ	10^{-4}	-445.605419	-446.719032	2.8275	-446.810728	-446.685229	3.4150
		10^{-5}	-445.605518	-446.719128	2.8275	-446.810813	-446.685588	3.4075
Cl_4benzoquinone	aug-cc-pVDZ	10^{-4}	-2215.518993	-2216.773026	2.0570	nc	nc	nc
		10^{-5}	-2215.519049	-2216.773079	2.0569	nc	nc	nc
	cc-pVTZ	10^{-4}	-2215.748130	-2217.258070	2.0250	nc	nc	nc
		10^{-5}	-2215.748718	-2217.258650	2.0252	nc	nc	nc
F_4benzenedicarbonitrile	aug-cc-pVDZ	10^{-4}	-810.306689	-811.829601	1.3550	nc	nc	nc
		10^{-5}	-810.306728	-811.829646	1.3546	nc	nc	nc
	cc-pVTZ	10^{-4}	-810.597898	-812.401006	1.3918	nc	nc	nc
		10^{-5}	-810.598102	-812.401200	1.3918	nc	nc	nc
F_4benzoquinone	aug-cc-pVDZ	10^{-4}	-775.346807	-776.706667	1.8600	nc	nc	nc
		10^{-5}	-775.346838	-776.706696	1.8601	nc	nc	nc
	cc-pVTZ	10^{-4}	-775.630995	-777.257213	1.8086	nc	nc	nc
		10^{-5}	-775.631166	-777.257365	1.8088	nc	nc	nc

References

[1] A. Shaalan Alag, D. P. Jelenfi, A. Tajti and P. G. Szalay, *J. Chem. Theory Comput.*, 2022, **18**, 6794–6801.

[2] J. W. Knight, X. Wang, L. Gallandi, O. Dolgounitcheva, X. Ren, J. V. Ortiz, P. Rinke, T. Körzdörfer and N. Marom, *J. Chem. Theory Comput.*, 2016, **12**, 615–626.

[3] R. M. Richard, M. S. Marshall, O. Dolgounitcheva, J. V. Ortiz, J.-L. Bredas, N. Marom and C. D. Sherrill, *J. Chem. Theory Comput.*, 2016, **12**, 595–604.

[4] M. Gałyńska, P. Tecmer and K. Boguslawski, *J. Phys. Chem. A*, 2024, **128**, 11068–11073.
